# Exploring ion-ion preferences through structure-property correlations: amino acid-derived, bis(guanidinium) disiloxane salts

**DOI:** 10.1038/s41598-020-57539-0

**Published:** 2020-01-20

**Authors:** Łukasz Tabisz, Zbigniew Rozwadowski, Andrzej Katrusiak, Bogusława Łęska

**Affiliations:** 1 Faculty of Chemistry, Adam Mickiewicz University in Poznań, Uniwersytetu Poznańskiego 8, Poznań, 61-614 Poland; 2 Faculty of Chemical Technology and Engineering, Department of Inorganic and Analytical Chemistry, West Pomeranian University of Technology, Piastów 42, Szczecin, 71-065 Poland

**Keywords:** Analytical chemistry, Organic chemistry, Physical chemistry, Supramolecular chemistry, Chemical synthesis

## Abstract

In a more synthetical approach to the study of ion-specific phenomena, four dipodal bis(guanidinium) siloxanes have been synthesized starting from glycine, β-alanine, γ-aminobutanoic acid, L-proline and 1,3-bis(3-aminopropyl)tetramethyldisiloxane. Together with their non-amide progenitor they were comparatively studied in regards to their interactions with nine different anions: sulphate, chromate, molybdate, benzoate, chloride, azide, nitrite, nitrate and thiocyanate. Their aqueous solubilities, form, ^1^H NMR and FT-IR spectra were examined while searching for anion-specific interactions falling in- or outside of the Hofmeister series. We show that although the “chao-” and “kosmotropic” ions affect the properties of solutions in a predictable way, more selective cation-anion pairing is responsible for phase separation and crystallinity. As a prominent example, crystal structure of one of the benzoate salts was successfully obtained and reveals a synergy of hydrophobic packing, ionic and hydrogen bonding. Immobilized but still flexible siloxane bridges give rise to crystals described by *P* 4_2_/n space group and neatly segregated into hydro- and lipophilic sections.

## Introduction

The ionic bond is an interaction that is both elemental and peculiar. In stark contrast to covalently bound atoms (and despite comparable energies being involved) pairs of cations and anions can seldom be called a single molecule. Extremely dynamic, strictly charge-dependent and non-directional nature of that interaction has made it, traditionally, a poor choice for designing molecular receptors with high selectivity. However, one instance in which “ion-specific interactions” are referred to on a regular basis is the Hofmeister series, a classification based on their ability to salt in or salt out proteins^1^. But this usage of the term can sound somewhat contradictory to a supramolecular chemist - after all, can’t the interactions *either* be truly specific *or* always follow the same, well-established trend?

One can of course argue that this “trend” has eluded satisfactory theoretical explanation for well over a century^2^ – but recently the interplay between water structure, ions, macromolecular solutes and physicochemical properties of the solution is becoming, again, a widely debated subject^3–5^. Accordingly, the number of papers claiming to explain - at least in part - what is already known from experimental data has risen sharply^6–8^. With these attempts to establish an underlying cause came numerous exceptions to the rule - cases which a supramolecular chemist would be much more inclined to call “specific”. One interesting example is the “reverse Hofmeister”^9^, observed for some (less common) positively charged proteins, e.g. lysozyme^10^. This seems logical at first glance: anions with high charge density, usually restricted (in case of negatively charged polypeptides) to bulk water can now approach the cationic moieties and instead compete with native intramolecular interactions, promote the unfolding and “water-mixing” of the macromolecule. Conversely, however, research concerning the impact of anions on the salting in (or out) of ionic liquids did not show a reversed, but only a slightly altered (if at all) trend^11–13^. And as many other papers report inconsistent or downright conflicting findings^14–16^, perhaps it is time to move past treating Hofmeister as the universal reference, discern between solvation/structuring effect of ions and their *truly* selective interactions, and establish new experimental trends - aiding theoretical studies in breaking down influences from individual functional groups, their spacing and cooperation. It was our belief with the present study that valuable information could be obtained from comparative analysis of series of compounds: complicated enough to include more than one functionality, yet simple enough to allow for differentiation of their individual impact on bulk physicochemical properties.

Starting with the abovementioned lysozyme/ionic liquid behaviour discrepancy and wanting to also investigate the structuring effect of anions on the separating organic phase, we decided to synthesize amino acid-linked bis(guanidines), in which the polar moieties are separated by a siloxane bridge. This idea stemmed from a previous study, which revealed an abnormally low water solubility of the prepared 1,3-bis(3-guanidinopropyl)-tetramethyldisiloxane nitrate (parent chloride was fully water-miscible)^17^. Addition of amide bonds seemed very attractive, as they often co-exist with guanidine moieties in many polypeptides and are a key ingredient in their higher-than-primary structure. Guanidines, on the other hand, have well-established “chaotropic” properties in their own right^18^. Siloxane bridges, though fairly chemically and very thermally resistant, impart a strong tendency for disorganization and non-crystallinity on the molecule, which stems from the extremely low energy barrier for rotation around the Si-O bond^19^. This results in most siloxanes presenting as liquids, oils or waxy solids at room temperature (a point of connection with ionic liquids). The apparent crystallinity of the abovementioned nitrate was yet another reason for choosing that particular class of compounds.

Apart from the obvious advantage of easily comparable results, one of the drawbacks of articles referring to Hofmeister series as a common denominator is the limitation of studied anions to those with well-established places on that spectrum: phosphate, sulphate, halides, nitrate, perchlorate and thiocyanate. In an effort to find more subtle differences, we expanded our search. For example, while the charge density difference between sulphate and perchlorate explains why these two geometrically similar oxoanions exert significantly different influences on water solutions, we chose chromate and molybdate (same geometry and charge) instead. Azide, an ion very rarely studied in this context, was also included - due to its specific linear geometry (similar to thiocyanate) and significant electronic contribution from the characteristic ^−^N = N^+^ = N^−^ resonance structure.

## Results and Discussion

### Design and synthesis

The intramolecular hydrogen bonding between amide moieties determines the α-helical or β-sheet structure of a peptide chain; we theorized that in smaller molecules, amide-guanidine hydrogen bonds could also play an “organizing” role, and possibly even compete with anions for access to nitrogen-bound hydrogens. Therefore, apart from glycine, β-alanine and γ-aminobutanoic acid were chosen as linkers; despite not being proteinogenic, both play important roles in the human body. The final amino acid - proline - was chosen as it can fit both at the beginning (carbonyl-guanidine distance, strain) and the end (number of carbon atoms, lipophilicity) of the series. Being a secondary amine, with sterically-hindering ring, it shows severe restrictions in the Ramachandran plot - in contrast to glycine, the least restricted amino acid in terms of ψ and ϕ angles^20^.

**Figure 1:**
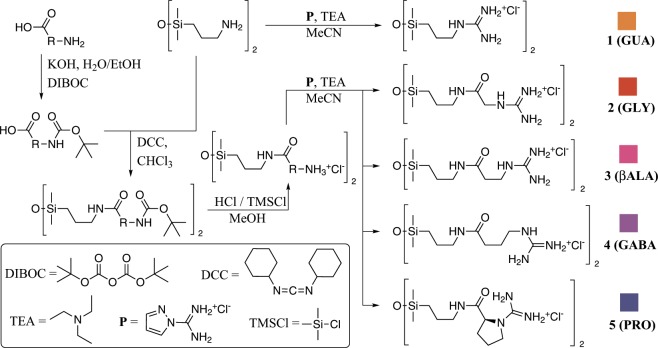
Synthesis of five bis(guanidinium) disiloxane chloride salts. Please refer to Section S2 in the Supplementary Information for detailed procedures.

The syntheses utilized standard reagents and pathways to obtain symmetrical diamides with tetramethyldisiloxane backbone and guanidine terminal groups (Fig. 1). At later stages no salts other than chlorides were used to ensure anionic purity of samples (e.g. drying was performed using anhydrous ethanol). Non-amide bis(guanidinium) salt, 1,3-bis(3-guanidinopropyl)-tetramethyldisiloxane dichloride (**GUA**) was prepared as described before^17^. The residual solvent had to be removed by repeated lyophilisation; all compounds were obtained as pale yellow oils or semisolids and no crystallization was observed after 3 months in 4 °C. All synthesized dichlorides were fully miscible with water at ambient temperature.

### Anion-exchange and solubility studies

**Figure 2:**
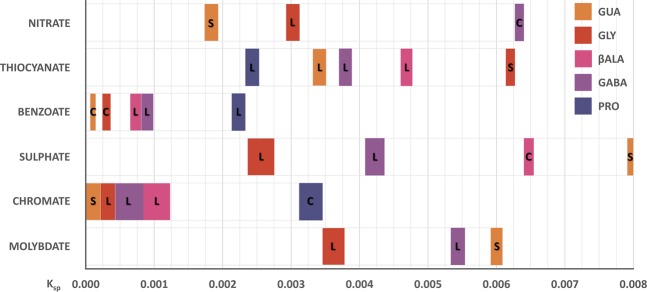
Aqueous solubilities of different salts of bis-guanidinium diamides (estimated as solubility product constant range, uncorrected). Letters denote the separating organic phase: L - liquid, C - “crystallizing” (solidifying liquid phase or crystallization from oil during first 24 h in 4 °C), S - immediate solid precipitate.

Preparation of different salts was combined with the study of their aqueous solubility and form. Solubility product constants were estimated on the basis of incremental addition of sodium salt solutions to samples containing 0.1 mmol of disiloxane. Molar concentrations of ions corresponding to the increments preceding and resulting in the permanent clouding of solution (or outright precipitation) were used to calculate the K_sp_ range (Fig. 2, for experimental details see Section S3 in Supplementary Information).

Apart from yellow chromates, all isolated salts were colourless liquids or solids. Salts containing monovalent anions were found to be very soluble in methanol, ethanol and dimethyl sulfoxide (DMSO), while sulphates, chromates and molybdates were only soluble in water, barely soluble in DMSO and insoluble in alcohols. No azides separated in the studied concentration range, and only the nitrite of **GUA** precipitated (solid, K_sp_ range: 5.26-5.42·10^−3^). This last dication was indeed the most prone to phase separation, in contrast with **PRO**, of which only thiocyanate, benzoate and chromate were obtained in such way. Based on those results, we theorized that there are two distinct mechanisms at play: pure lipophilic pairing, with no meaningful interactions, which was evident for thiocyanate (according to calculations, **GUA** is even less hydrophilic than **PRO** - although the aliphatic ring and lack of one guanidinium proton must have an additional, detrimental effect on hydration, thus promoting separation). The reverse trend for the rest of studied anions can only be explained by favourable cation-anion interactions, counteracting the dissociative effect of water. If the anion needs only the guanidine moieties to form strong intermolecular interactions, **GUA** appears at the low K_sp_ end of the trend. If the anion utilizes amide protons, **GUA** instead shows up at the high K_sp_ range. The only other observable exception – switching of **βALA** and **GABA** – could be traced to the most favourable intramolecular (6-membered ring) hydrogen bonding between carbonyl and guanidinium -N**H**- protons in the former. If the anion has to compete for that interaction, the overall “polymeric salt” structure can effectively be less preferable. Furthermore, benzoate is the only counterion for which the regular arrangement of diamides is retained – this observation and the notion that the carboxylic group binds strongly and selectively with two –NH_2_ guanidinium groups (not intramolecularly hydrogen-bonded), in a motif known as a “salt bridge”^21^ in proteins, firmly enforce each other. The extremely low solubility ofchromates is also interesting; chromate is the middle-sized of the three studied tetrahedral dianions, but sulphates and molybdates behaved fairly similar. It is possible that spacing of oxygens in chromate fits bestthe guanidinium cation, but the key factor is probably the very high polarization of Cr-O bond (difference in Pauling electronegativity of 1.88), which strengthens the hydrogen bonds that are formed.

### Comparison of ^1^H NMR spectra

**Figure 3:**
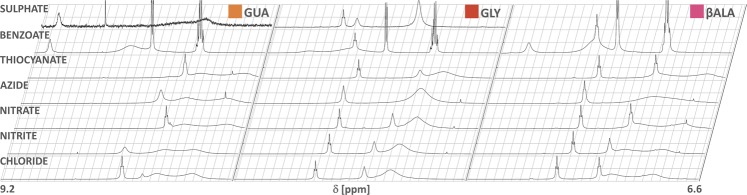
Comparison of ^1^H NMR spectra of **GUA**, **GLY** and **βALA** salts in DMSO-d6 (nitrogen-bound protons, 6.6–9.2 ppm). Only sulphates of **GLY** and **GUA** were sufficiently soluble to produce well-defined signals (shown as per saturated solution; other salts at 0.05 M). Table 1 summarizes all the chemical shifts (and shapes) of relevant peaks.

Although not ideal, in order to study exchangeable protons, DMSO-d6 was used in place of water in NMR experiments. It has been established that DMSO behaves similarly under the influence of different ions (i.e. the physicochemical properties of both solvents change in accordance with the same lyotropic series)^22,23^. While most bisguanidines were studied as 0.05 M solutions, unfortunately only the sulphates of **GUA** and **GLY** were sufficiently soluble to produce informative signals (Fig. 3) and spectra of their saturated solutions are presented instead. Details for all salts are summarized in Table 1.

**Table 1:** ^1^H NMR signals of N-H protons in different salts of bis(guanidinium) disiloxanes (DMSO-d6, 400 MHz, 0.05 M).

Salt	^1^H shifts [ppm], multiplicity, integration				Salt	^1^H shifts [ppm], multiplicity, integration			
	-(O = )C-NH-	-NH-	-NH_2_			-(O = )C-NH-	-NH-	-NH_2_	
GUA-Cl	—	7.82, t, 1 H	7.42, br, 2 H	7.01, br, 2 H	βALA-NO_3_	8.06, t, 1 H	7.49, t, 1 H	7.43, br, 2 H	6.89, br, 2 H
GUA-NO_2_	—	7.86, br^[c]^, 1 H	7.30, br, 2 H	7.06, br, 2 H	βALA-N_3_	8.09, t, 1 H	(7.7-6.7) 7.29, vbr^[c]^, 5 H		
GUA-NO_3_	—	7.46/7.42, t, 1 H (5:1)	7.25, br, 2 H	6.76, br, 2 H	βALA-SCN	8.00, t, 1 H	7.35, t, 1 H	7.32, br, 2 H	6.77, br, 2 H
GUA-N_3_	—	7.60, br^[c]^, 1 H	7.31, br, 2 H	6.83, br, 2 H	βALA-Bz^[d]^	8.11, t, 1 H	8.89, br^[c]^, 1 H	(8.4-7.9) ~8.13, br, 4 H	
GUA-SCN	—	7.40, t^[b]^, 1 H	7.20, br, 2 H	6.71, br, 2 H	GABA-Cl	8.01, t, 1 H	7.88, t, 1 H	7.46, br, 2 H	7.03, br, 2 H
GUA-Bz^[d]^	—	9.04, br^[b]^, 1 H	(8.3-7.7) ~8.11, vbr, 4 H		GABA-NO_2_	7.96, t, 1 H	7.90, t^[b]^, 1 H	7.46, br, 2 H	7.09, br, 2 H
GUA-SO_4_^[a]^	—	9.02, br^[b]^, 1 H	(8.0-7.0) ~7.32, vbr, 4 H		GABA-NO_3_	7.93, t^[b]^, 1 H	7.58, t^[b]^, 1 H	7.32, br, 2 H	6.85, br, 2 H
GLY-Cl	8.28, t, 1 H	7.72, br^[b]^, 1 H	7.42, br, 4 H		GABA-N_3_	7.98, t, 1 H	(7.8-6.6) 7.33, vbr^[c]^, 5 H		
GLY-NO_2_	8.20, t, 1 H	7.68, br^[c]^, 1 H	7.39, br, 4 H		GABA-SCN	7.91, t, 1 H	7.47, t, 1 H	7.30, br, 2 H	6.78, br, 2 H
GLY-NO_3_	8.16, t, 1 H	7.55, br^[br]^, 1 H	7.26, br, 4 H		GABA-Bz^[d]^	8.08, t, 1 H	9.21, t^[b]^, 1 H	(8.3-7.7) ~8.05 vbr, 4 H	
GLY-N_3_	8.19, t^[b]^, 1 H	(7.0-7.6) ~7.30, br^[c]^, 5 H			PRO-Cl	8.32, t, 1 H	—	7.47, br, 4 H	
GLY-SCN	8.09, t, 1 H	7.39, br^[c]^, 1 H	7.12, br, 4 H		PRO-NO_2_	8.12/8.07, t, 1 H (3:1)	—	7.39, br, 4 H	
GLY-Bz^[d]^	8.21, t, 1 H	8.73, br^[c]^, 1 H	8.26, br, 4 H		PRO-NO_3_	8.07/8.02, t, 1 H (3:1)	—	7.25, br, 4 H	
GLY-SO_4_^[a]^	8.42, t, 1 H	8.27, br^[c]^, 1 H	7.56, br, 4 H		PRO-N_3_	8.10/8.06, t, 1 H (3:1)	—	7.26, br, 4 H	
βALA-Cl	8.18, t, 1 H	7.69, t, 1 H	7.56, br, 2 H	7.08, br, 2 H	PRO-SCN	7.97, t, 1 H	—	7.11, br, 4 H	
βALA-NO_2_	8.07, t, 1 H	7.65, br^[b]^, 1 H	7.59, br, 2 H	7.03, br, 2 H	PRO-Bz^[d]^	8.48, t, 1 H	—	(9.3-7.6) ~8.40, vbr, 4 H	

t -triplet, br - broad, vbr - very broad (>0.5 ppm). ^a^Due to low solubility, ^1^H NMR spectra of sulphates were recorded for the highest possible concentration instead of standard 0.05 M. ^b^Poorly resolved (t) or not visibly resolved (br) peak, but coupling evident in the neighboring -CH_2_- signal (presents as quartet). ^c^No related coupling observed in the neighbouring -CH_2_- signal (presents as triplet). ^d^ – Benzoate salt.

**Figure 4:**
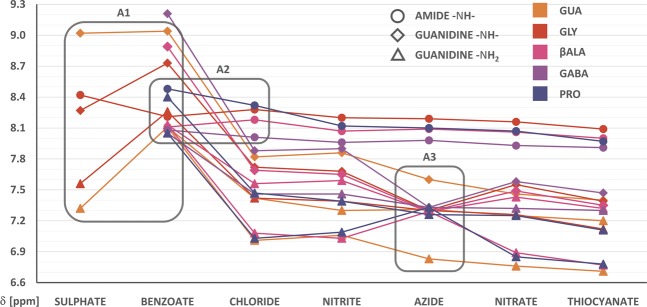
Effect of counterion on the chemical shifts of nitrogen-bound hydrogens in bis(guanidinium) diamides. Anions were arranged as per standard Hofmeister series, with the exception of benzoate. Regions of the most interesting anomalies are indicated by grey brackets (A1-3).

While the previous experiment elucidated much about trends arising from the structure of the organic cation, anion-based trends become more visible in ^1^H NMR spectra. Signals from labile protons shift steadily upfield as the counterion is changed from sulphate to thiocyanate. While it was proved that the chemical shifts of solvent’s atoms can change in the presence of ions in a manner not directly translatable to Hofmeister series rationale^24^, the solvent-mediated impact on other solutes remains in agreement with the notion that poorly solvated anions render the polar solvent less aggressive^25^ (by actually allowing it to retain its typical, hydrogen-bonded structure or, in the case of DMSO, one based on other strong dipole-dipole interactions). It follows that a sufficiently selective ion-ion interaction would be visible as a deviation from the uniform changes brought about by the dominant solvent-ion interactions. Anions derived from weak acids (azide, benzoate and - to a lesser extent - nitrite) produced some aberrations in the trend, and their relative arrangement sometimes changed, depending on the organic cation. However, a closer look at those anomalies (denoted **A1-3** in Fig. 4) calls into question whether they can be fully ascribed to the basicity of the anion. Benzoate’s preference towards guanidinium hydrogens can be easily seen by tracing the chemical shifts in **GLY** from chloride to sulphate: while the amide proton is actually shifted upfield in benzoate (compared to other two), guanidinium protons are shifted heavily downfield. Sulphate, on the other hand, doesn’t discriminate against amide hydrogens (**A1**). On the contrary, the chemical shift of guanidinium -N**H**- in **GUA-SO**_**4**_ is almost the same as in **GUA-Bz** (benzoate salt), but is much lower in **GLY-SO**_**4**_, where the alternative - amide - binding site is present (and the relevant amide proton signal is, in fact, shifted fairly downfield, corroborating the presence of, on average, stronger hydrogen bonds around this moiety). This is in accordance with our reasoning concerning the different trends seen in aqueous solubilities of salts. The second irregularity (**A2**) is more subtle, as it concerns only amide protons - far less affected by anion substitution, but also less prone to deviations in general. However, for **GLY** and **βALA**, the chemical shift of the amide proton in benzoate is notably smaller than in chloride salt, while for **GABA** and **PRO** the situation is reversed. This can be explained by strong interactions of the carboxylate anion interfering with internal structure (and, therefore, relative location of amide hydrogen), important in the former two cations, but less so in the longest molecule, **GABA**. Along this line, **PRO** can be presumed to be too constrained for the amide proton to be effectively dislocated - and that observation, together with extremely similar chemical shifts of guanidine -N**H**_**2**_ protons in salts of **GLY** and **PRO** with poorly hydrogen-bonding anions, is a warning not to extrapolate a few similarities (in this case: merged -N**H**_**2**_ signals, guanidine-carbonyl distance) to the entirety of intra- and intermolecular behaviour of a molecule.

The last registered, unmistakable anomaly was exclusive to the azide salts; in all cations with an amide linker, this counterion caused the merging of all signals from guanidinium protons and the disappearance of related coupling in the neighbouring -C**H**_**2**_- peak (**A3**). At first we presumed the basicity of N_3_^−^ was high enough to greatly facilitate proton exchange throughout the guanidinium moiety. However, when we recorded the spectrum for **GUA**, despite the disappearance of coupling (indicating fast exchange), all three signals remained separate. Although we believe this proves that another truly ion-specific interaction is at play here (and underlines the importance of discerning between decoupling, fast exchange and merging of signals when studying ionic interactions by means of ^1^H NMR)^26,27^, we did not find a reliable explanation for why the presence of an amide bond would assist in equalization of guanidinium protons in the presence of azide. Possibly, N_3_^−^ acts as a bridge between the two moieties, opening an additional pathway of proton transfer throughout the molecule. More specific research is probably called for, but was beyond the scope of presented work.

### FT-IR spectra of thin films

The siloxane salts separating from aqueous solution were rarely solid, let alone crystalline - as is the case with many precipitating proteins, polymers, surfactants and other flexible molecules of mixed polarity. Of the many possibilities for sample preparation for FT-IR spectroscopy, we therefore decided on thin films as best approximation of the partially disorganized state of the organic phase when it partitions from solution. A comparative spectrum of **GUA-NO**_**3**_ obtained in KBr tablet validated this approach, and highlights how different degree of matter organization can immensely impact the observable interactions – even between fairly simple molecules or ions (Table 2).

**Table 2:** FT-IR data for five most common, discernible bands in spectra of bis(guanidinium) diamides (thin films obtained by evaporation of EtOH solutions).

Salt	N-H ν [cm^−1^]			C = X ν [cm^−1^]	Amide II band [cm^−1^]	Salt	N-H ν [cm^−1^]			C = X ν [cm^−1^]	Amide II band [cm^−1^]
	I	II	III	IV			I	II	III	IV	
GUA-Cl	3332	3260	3164	1661	—	βALA-N_3_	3320sh	3272	3168	1651	1557
GUA-NO_3_	(sh)^[b]^, 3343	3269	3177	1661		βALA-SCN	3326	3272sh	3174	1653	1560
GUA-NO_3_^[a]^	3396^[b]^, 3340	3279	3202	1667, 1629^[b]^	—	βALA-Bz	(sh)	3272	3174	1653	1546
GUA-N_3_	3358	(sh)	3152	1661	—	GABA-Cl	(sh)	3269	3158	1650	1551
GUA-SCN	3429	(sh)	3195	1645	—	GABA-NO_3_	(sh)	3273	3161	1650	1550
GUA-Bz	3339	3270	3159	1678-1633^[c]^	—	GABA-N_3_	(sh)	3269	3164	1650	1555
GLY-Cl	3284sh	3263	3159	1659	1559	GABA-Bz	(sh)	3269	3164	1650	1548
GLY-NO_3_	3307sh	3271	3174	1659	1555	PRO-Cl	3306	3205	3143	1650, 1613^[b]^	1539
GLY-N_3_	3307sh	3268	3174	1659	1559	PRO-NO_3_	3310	3210	3148	1651, 1613^[b]^	1539
GLY-SCN	3326	(sh)	3174	1657	1562	PRO-N_3_	3315	3218	3151	1655, 1613^[b]^	1539
GLY-Bz	(sh)	3266	3164	1663	1544	PRO-SCN	3410	(sh)	(sh)	1636	1537
βALA-Cl	3320sh	3268	3159	1651	1555	PRO-Bz	3310	3243	3148	1660, 1613^[b]^	1549

^a^Spectrum recorded in KBr tablet. ^b^Additional band, not discernible in most cases. ^c^Broad, jagged band, without a dominating maximum in denoted range. (sh) Appears only as a shoulder on the nearest band; wavenumber instead of brackets is given only when the maximum could be pinpointed with reasonable accuracy.

**Figure 5:**
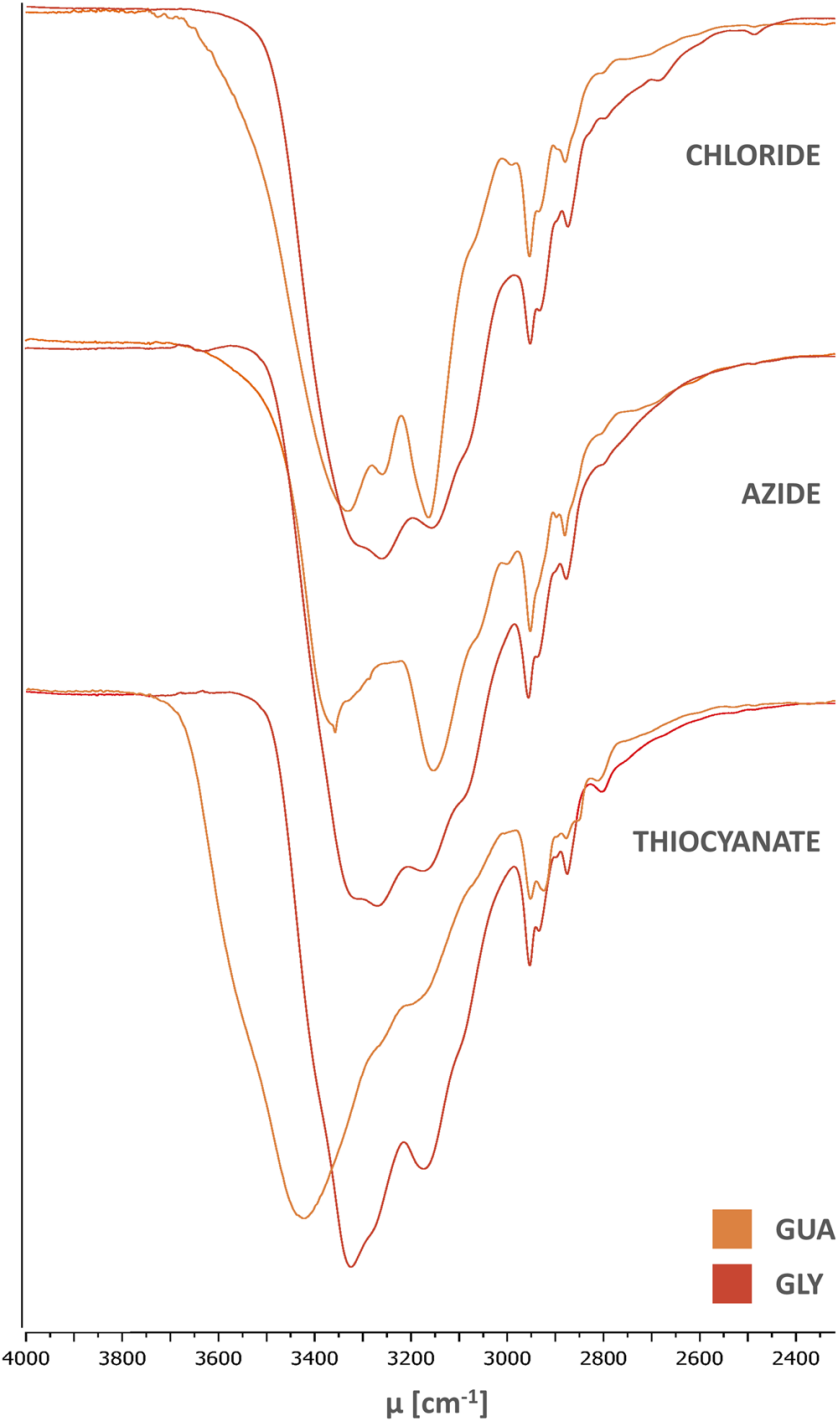
Comparison between FT-IR spectra (stretching X-H vibrations) of bis(guanidinium) salts with anions of decreasing hydrogen-bonding capabilities. Due to the lack of an amide moiety, **GUA** is far more affected by the lack of a good hydrogen bond acceptor, and generally more susceptible to counter-ion influences on IR absorptions.

**Figure 6:**
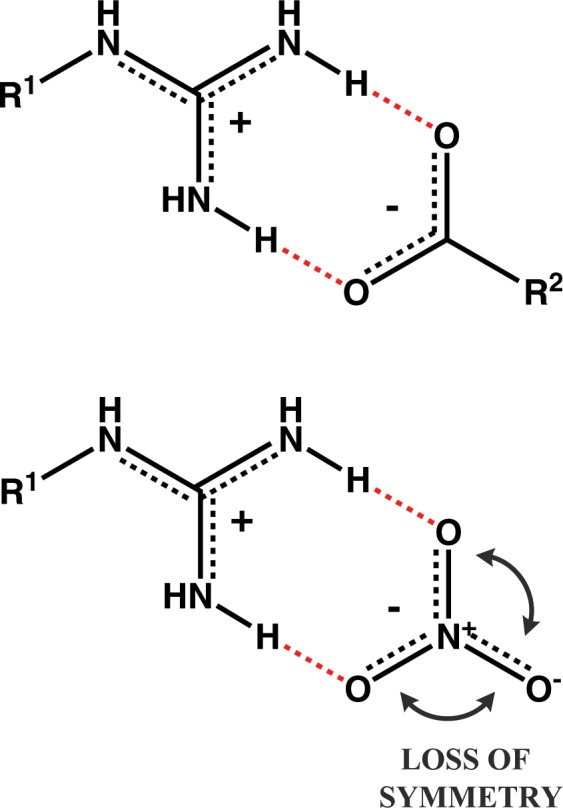
Comparison between spatial arrangement of a guanidinium cation and a carboxylate anion in a “salt bridge” (motif combining ionic and hydrogen bonding) and an analogous arrangement with a nitrate ion. Note that in the latter, one nitrogen-oxygen bond in the anion becomes dissymmetrized from the rest.

The complementarity of FT-IR investigations is revealed by careful study of the differences between rather similar spectra of disiloxane salts. The most evident is the thiocyanate, with nearly universal disappearance of some individual bands in the N-H region and moving of rest to higher wavenumbers, indicating weaker hydrogen bonding. Particularly, the **II** band can only be pinpointed for **βALA-SCN**, another sign of noticeably stronger intramolecular bonding “substituting” for the anion due to favourable carbonyl-guanidinium distance. Furthermore, in **GUA**, where no intra- or intermolecular hydrogen bonding utilizing amide moieties can take place, a similar situation is also seen for the azide salt (Fig. 5). Much larger differences in band positions (and shapes) observed for salts of that cation further strengthen the notion that the amide group has a “buffering” effect on the molecule - or rather the whole organic phase.While in solution nitrate behaved very much according to its place in the Hofmeister series, in thin films it becomes evident that this particular anion also has some degree of affinity for guanidinium (albeit less so than the carboxylic group). This manifests through the frequencies in nitrates being on par or even lower than those in azides (bands **I-III**) or even chlorides (**amide II band** – however, it is worth noting that usually that band’s frequency rises with stronger intermolecular hydrogen bonding^28^; in our case, only with **PRO** this trend wasn’t perfectly reversed). In solid **GUA-NO**_**3**_ (in KBr) two more absorption bands could be discerned, and most other were, understandably, much sharper than in thin films. However, it was the inherent nitrate stretching absorption band that has changed most dramatically, prompting us to analyse that specific aspect of FT-IR spectra more closely. It is well known that changes in absorption intensity can be indicative of strong interactions (e.g. hydrogen bonding)^29^, even if not accompanied by important frequency shifts. For a trigonal anion such as nitrate, only the asymmetric stretch is IR-active, and entails the shortening of one N-O bond, while other two become elongated. If we assume the nitrate-guanidinium ion pair forms analogously to carboxylate-guanidinium (Fig. 6), the difference in partial charge and distance between two oxygens involved in the bonding and the third, unused, would increase the change in dipole moment on passing from ground to excited state^30^. That change is directly translatable to intensity of absorption, as was indeed found in our compounds (Table 3).

**Table 3:** Nitrate-specific, out-of-phase stretching vibration in different bis(guanidine) samples studied by FT-IR.

Compound	N-O ν [cm^−1^]	N-O/Si-O^[a]^ ν Intensity ratio	N-O/Si-O ν I·HW^[b]^ ratio
GUA (KBr)	1384	2.28	69
GUA (thin film)	1392	6.14	458
GLY	1372	2.10	256
βALA	1388	1.32	194
GABA	1361	3.52	297
PRO	1354	1.94	144

^a^Si-O stretching band was chosen as a reference due to stability of its shape and minimal overlapping. ^b^Intensity multiplied by band width at half height.

Whether we look at pure intensities or take the band widening into account, it is obvious that **GABA** interacts strongly with nitrate, next only to **GUA**. This highlights how important the spacing between different moieties in a molecule can be (even a very flexible one, such as siloxane) and again reinforces the given explanation for **βALA** salts being much less prone to precipitation. The reason for such a colossal difference in the presentation of nitrate absorption band (in thin films and in solid - recrystallized - sample in a KBr tablet) can be traced to the level of matter organization examined in both approaches. It is improbable that in the separating, liquid organic phase (approximated by thin films) the full potential of possible hydrogen networking is utilized - and it is just as unlikely that for such compounds as those studied, anything less could force them to adopt a crystalline form. Therefore the strongest, asymmetric bonding with only two nitrate oxygens dominates in non-crystalline, while in solid/crystalline phase all three oxygens must be fairly uniformly hydrogen bonded to guanidinium moieties (see Fig. 6 and subsequent section).

### Crystal structures

**Figure 7:**
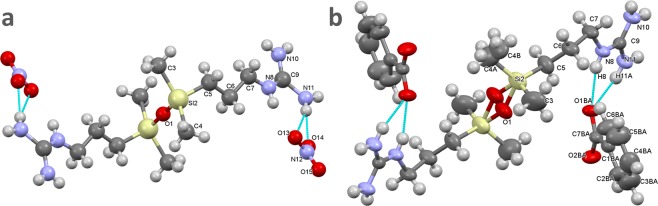
Structures determined by X-ray diffraction of two symmetry-independent units of bis(guanidinium) salts. (**a**) **GUA-NO**_3_ shown as a model of balls-and-sticks of arbitrary sizes; and (**b**) **GUA-Bz** with thermal ellipsoids shown at the 50% probability level and one set of disordered atoms O1 and C4 (in the second unit this disorder is ignored for clarity). Atomic labels of one asymmetric unit have been shown for non-H atoms.

**Figure 8:**
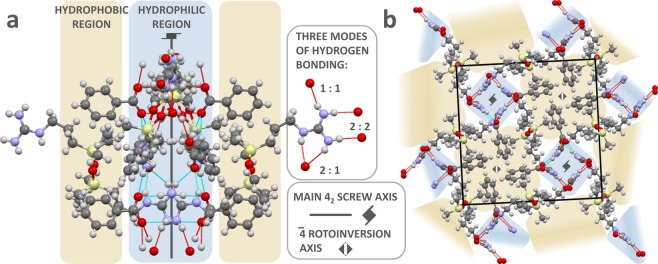
Fragment of crystal structure of benzoate salt of **GUA**, showing neatly separated hydrophilic and hydrophobic sections. (**a**) View perpendicular to main 4_2_ screw axis (grey), (**b**) view along the [z] axis. Cyan and red dashed lines show the extent of hydrogen bonding. Silicon atoms are coloured yellow, oxygen - red and nitrogen - pale violet.

Ammonium siloxane compounds without sterically hindering groups are known for their reluctance to form single crystals^31^. This feature can be connected to the absence of strong cohesion forces and excessive conformational flexibility of the Si-O-Si bridge^19^ of the molecular cation. However, we succeeded in obtaining single crystals of two salts of the series, which elucidated the characteristic structural and conformational features, as well as the aggregation capabilities of these compounds. Reiterated crystallizations from various solvents and using different methods (solvent evaporation, lowering temperature, diffusion) of precursor salt **GUA-NO**_**3**_ finally yielded best single crystals from 20% aqueous ethanol evaporated slowly at ambient temperature. Apart from the very small size of the crystals, allowing for only the strongest reflections to be measured reliably, these triclinic crystals (Table 4) turned out to be very strongly structurally disordered and this determination was continued only as a structural verification of the chemical formula. Although the non-centrosymmetric model could yield lower R factors (R1 below 0.20), its refinements were unstable and we accepted the space group *P* $\bar{1}$, for which the course model could be refined without any constraints. In this model the oxygen atom is located at an inversion centre and half of the dication is symmetry independent (Fig. 7a), but the huge atomic displacement parameters evidence the disorder of the structure. This disorder has also been observed in the **GUA-Bz** crystal – albeit of much better quality (obtained from 30% aqueous ethanol in −15 °C, after 4 days) and characterized by *P* 4_2_/n space group. The quality of crystals and of the X-ray diffraction data was sufficient to allow for reliable anisotropic refinement of the structure, with the H-atoms located from the difference Fourier maps. A reliable structural model with H-sites consistent with the bond lengths of their carriers and with the H-bonding pattern has been obtained. While the dication is again located on a centre of inversion, the split positions of atoms O1 and methyl C4 can be clearly discriminated (Fig. 7b). The Si2-O1-Si2′ angle is 150.9°, and the disordered O1–O1′ sites are 0.83 Å apart. This feature connects to two sites, 0.67 Å apart, of disordered methyl C4. In this structure, the guanidine moieties are fivefold NH–O hydrogen bonded to three benzoate anions; benzoate oxygen O1BA is a 3-fold H-acceptor and O2BA is a 2-fold H-acceptor (see Figures S4.1.3 and S4.1.4 in Supplementary Information for close-ups with bond dimensions). Noteworthy, such bifurcated NH–O bonding between guanidinium and oxoanions is relatively rare compared to the pair of NH–O bonds linking two -NH_2_ groups with both the oxygen atoms. In the Cambridge Structural Database^32^ there are currently 368 crystal-structure deposits with this type of bifurcated bonds. Such bonding prevails particularly in structures of small ions, notably (for the sake of this discussion) in guanidinium nitrate^33^. This highly symmetrical H-bonding pattern, resulting in honey-comb layers, could be destabilized only by high pressure (exceeding 200 MPa), when one of the NH–O bonds is broken and a bifurcated H-bond is formed in its stead^34^, - a similar one to that present in **GUA-Bz**. Such bifurcated bond is flexible (compared to the NH–O bonding pairs) and allows a considerable rotation of the H-bonded moieties (compare Figs. 7 and 4 in Reference ^34^). It can be observed that this freedom of rotation is essential for the molecular aggregation even in the crystals of much simpler guanidinium benzoate^35^. The 4_2_ axes, indicated in the space group’s name, are located inside helices constructed from those guanidinium and carboxylate moieties (Fig. 8a). Every such cluster of ionic groups is in turn surrounded by four apolar regions, containing aromatic rings and non-disordered siloxane methyl groups pointing inward. Symmetry viewed from the centre of those hydrophobic sections is described by a 4-fold rotoinversion axis. The inner crystal structure is therefore neatly segregated into hydrophilic and hydrophobic segments, with siloxane bridges and neighbouring alkyl chains acting as their boundaries (Fig. 8b). Together, these findings clarify that it was the cooperation between three major types of interactions: ionic bonds, hydrogen networking and hydrophobic effect that enabled the immobilization the inherently flexible siloxane molecules into a crystal lattice with long-range ordering and heightened symmetry. In the absence of such complementary forces - which can depend solely on the counterion present - crystallinity is unlikely. The apparently large contribution from hydrophobic packing (typically prominent in peptides and some macrocycles) in the obtained crystal structure underlines the importance of this factor for aqueous solubilities and other properties of smaller organic salts of mixed polarity.

**Table 4:** Selected crystal data for salts **GUA**-**NO**_**3**_ and **GUA**-**Bz** (detailed information are available in crystallographic information files, deposited with the Cambridge Crystallographic Database Centre, as Supplementary publications number CCDC 1886835-1886836; they are available free of charge from www.ccdc.cam.ac.uk).

Salt	GUA-NO_3_	GUA-Bz
Formula	C_12_H_34_N_8_O_7_Si_2_	C_26_H_44_N_6_O_5_Si_2_
Space group	*P* $\bar{1}$	*P* 4_2_/n
Unit cell:		
a (Å)	7.025(5)	18.8363(2)
b (Å)	7.408(4)	18.8363(2)
c (Å)	13.797(6)	9.1296(2)
α(°)	89.29(4)	90
β(°)	84.16(4)	90
γ(°)	61.91(6)	90
*V* (Å^3^)	629.6(5)	3239.24(10)
*Z*/*Z*’	1/0.5	4/0.5
*D*_x_ (g·cm^−1^)	1.2148	1.1830

The spatial arrangement of ions in **GUA-NO**_**3**_ (although not definite due to the tentative determination) along with previous crystallographic data^33^ corroborates the previously given explanation for large differences in FT-IR spectra of nitrates (i.e. crystalline state utilizing more symmetrical arrangement of ion-ion interactions, and thin films lacking in such uniformity). Furthermore, there are closely located voids, over 2.2 Å in diameter, in the **GUA-Bz** structure (see Figure S4.1.5 in Supplementary Information). Although too small to accommodate solvent molecules, they are located together with the benzoate rings in the same channels running down [z]. It appears that similarly arranged structures of **GUA** with other carboxylate anions could also be possible.

## Conclusions

Four new, guanidinium-appended tetramethyldisiloxane diamides were synthesized using glycine, β-alanine, γ-aminobutanoic acid and L-proline. Those symmetrical molecules are characterized by mixed polarity and high flexibility of the central siloxane bridge, which result in their superficial resemblance to ionic liquids – despite their double positive charge that is easily accessible for counterions. The effect of those – namely: sulphate, chromate, molybdate, benzoate, nitrate, nitrite, azide, chloride and thiocyanate, on relationships between structure, aqueous solubilities, ^1^H NMR, FT-IR spectra and crystallinity – was studied in depth. It was established that – in contrast to typical ionic liquids, most polymers and peptides – the precipitation of studied salts, along with their non-amide precursor, was driven chiefly by ion-ion interactions, and not anion-solvent interactions (Hofmeister-type salting out/in phenomena). For such highly charged, small and flexible molecules, the separation from aqueous solution as an organic phase was found to follow two trends, depending predominantly either on the strength of bonds formed between ions, or pure lipophilicity of the formed salt. The latter mechanism dominated only for the very poorly hydrogen bonding thiocyanate, resulting in proline-derived bis(guanidine) separating most easily. For other anions, evidence of strong, preferential bonding points especially to benzoate, nitrate and chromate, with the first yielding even single crystals from aqueous ethanol. This level of organization turned out to be possible due to the synergy of salt bridges (ion-ion and hydrogen bonding) and very pronounced hydrophobic effect, which resulted in segregation of internal structure into polar and non-polar compartments.

The inclusion of an amide moiety results in a “buffering” effect, with the precursor bis(guanidine) disiloxane salt behaving more erratically, and showing much more pronounced changes in spectra on anion exchange. Presence of an amide group was also responsible for sharp decrease in solubilities of sulphates and molybdates; other anions, especially the three mentioned before, show a marked selectivity towards the guanidinium moiety, with which they most probably form characteristic salt bridge-type connections.

We believe that the acquired body of data - especially the exceptions, noted specific guanidinium-anion interactions and the widespread non-adherence of studied compounds’ solubilities/precipitation trends to the Hofmeister series – reveals the persisting deficits in our understanding of ionic bonding as it affects small and middle-sized, charged organic molecules. Large literature gaps still exist in the spaces between simple inorganic salts, very poorly coordinating ionic liquids and macromolecules, including proteins.

Quite possibly, it is at those boundaries that truly ion-specific interactions can be sieved out from solvent structuring effects, dehydration of solute molecules and intramolecular interferences.

## Supplementary information

Supplementary Information.

## Data Availability

All data generated or analyzed during this study are included in this published article (and its Supplementary Information files). Readers are encouraged to direct any further requests to the corresponding author.
