# Supplementary material for: Exploring ion-ion preferences through structure-property correlations: amino acid-derived, bis(guanidinium) disiloxane salts
Source: Sci Rep. 2020 Jan 20;10:646. doi: 10.1038/s41598-020-57539-0 (PMC6971042; doi:10.1038/s41598-020-57539-0)
Supplement: Supplementary file 1 — Supplementary Information. [file 41598_2020_57539_MOESM1_ESM.pdf]

Scientific Reports

Electronic Supplementary Information

Exploring ion-ion preferences through structure-property correlations: amino acid-derived, bis(guanidinium) disiloxane salts

Łukasz Tabisz, Zbigniew Rozwadowski, Andrzej Katrusiak and Bogusława Łęska

Table of contents

S1. General information 4

S2. Synthesis and compound characterization 4

S2.1. Synthetic procedure 4

S2.1.2. Materials 4

S2.1.3. Amino acid protection 4

S2.1.4. Diamide synthesis 4

S2.1.5. Diamide deprotection 5

S2.1.6. Bis(guanidine) synthesis 5

S2.2. Compound characterization. 6

Table S2.2.1. ^{1}H NMR data: all disiloxane diamides (A-C). 7

Table S2.2.2. ^{13}C NMR data: disiloxane bis(guanidines) (C). 8

Table S2.2.3. Elemental analysis data: disiloxane bis(guanidines) (C). 8

S3. Salt metatheses and solubility studies 9

S3.1. Materials 9

S3.2. Estimation of aqueous solubilities / K_{sp} of salts 9

S3.3. Preparation of different bis(guanidinium) salts 9

Table S3.3.1. Comparison of melting points: GUA salts. 9

S4. Additional crystallographic data. 10

S4.1. Additional figures. 10

Figure S3.1.1 The NH---O bonded helix fragment of the GUA-Bz structure viewed down [z]. 10

Figure S4.1.2. The NH---O bonded helix fragment of the GUA-Bz structure viewed down [y]. 10

Figure S4.1.3. Close-up fragment of the NH---O bonded helix in GUA-Bz structure with denoted hydrogen bond angles and distances (cyan dotted lines). 11

Figure S4.1.4. All hydrogen bonds formed by the guanidinium moiety in GUA-Bz structure, with denoted bond angles and distances (green and red dotted lines). 11

Figure S4.1.5. The GUA-Bz structure viewed along the [z] axis. The void regions probed with spheres 1.0 Å in 0.1 Å steps are indicated in gold. This drawing can be viewed in 3-dimensions as an autostereogram.[3] 12

S5. Spectra glossary 13

S5.1 NMR spectra 13

Figure S5.1.1. ^{1}H NMR spectrum of compound 1A. 13

Figure S5.1.2. ^{1}H NMR spectrum of compound 1B. 13

Figure S5.1.3. ^{1}H NMR spectrum of compound 1C (GLY). 14

Figure S5.1.4. ^{13}C NMR spectrum of compound 1C (GLY). 14

Figure S5.1.5. 2D spectrum (^{1}H-^{13}C HETCOR) of compound 1C (GLY). 15
Figure S5.1.6. ^{1}H NMR spectrum of compound 2A. 15
Figure S5.1.7. ^{1}H NMR spectrum of compound 2B. 16
Figure S5.1.8. ^{1}H NMR spectrum of compound 2C (βALA). 16
Figure S5.1.9. ^{13}C NMR spectrum of compound 2C (βALA). 17
Figure S5.1.10. 2D spectrum (^{1}H-^{13}C HETCOR) of compound 2C (βALA). 17
Figure S5.1.11. ^{1}H NMR spectrum of compound 3A. 18
Figure S5.1.12. ^{1}H NMR spectrum of compound 3B. 18
Figure S5.1.13. ^{1}H NMR spectrum of compound 3C (GABA). 19
Figure S5.1.14. ^{13}C NMR spectrum of compound 3C (GABA). 19
Figure S5.1.15. 2D spectrum (^{1}H-^{13}C HETCOR) of compound 3C (GABA). 20
Figure S5.1.16. ^{1}H NMR spectrum of compound 4A. 20
Figure S5.1.17. ^{1}H NMR spectrum of compound 4B. 21
Figure S5.1.18. ^{1}H NMR spectrum of compound 4C (PRO). 21
Figure S5.1.19. ^{13}C NMR spectrum of compound 4C (PRO). 22
Figure S5.1.20. 2D spectrum (^{1}H-^{13}C HETCOR) of compound 4C (PRO). 22
S5.2 FT-IR spectra. 23
Figure S5.2.1. FT-IR spectrum of 1,3-bis(3-guanidinopropyl)-tetramethyldisiloxane dichloride (GUA). 23
Figure S5.2.2. FT-IR spectrum of 1C (GLY). 23
Figure S5.2.3. FT-IR spectrum of 2C (βALA). 24
Figure S5.2.4. FT-IR spectrum of 3C (GABA). 24
Figure S5.2.5. FT-IR spectrum of 4C (PRO). 25
References 26

S1. General information

The NMR spectra were recorded on BRUKER Avance III HD spectrometer at room temperature (20°C) operating at 400.2 MHz (^{1}H) and 100.6 MHz (^{13}C). The standard Bruker software was used for acquisition and data processing.
The FT-IR spectra were recorded using an IFS 66/s FT-IR spectrophotometer from Bruker, equipped with an MCT detector (125 scans, resolution 2 cm^{-1}).
Elemental analyses were performed using Vario EL III (Elementar) elemental analyser. Three separate measurements were performed for each sample.

S2. Synthesis and compound characterization

S2.1. Synthetic procedure

S2.1.2. Materials

All amino acids were obtained from *BULK POWDERS UK* as nutritional supplements. Other chemicals were purchased either from *Merck* or *Avantor*, except the organosilicon precursor diamine (1,3-bis-(3-aminopropyl)-tetramethyldisiloxane), which was obtained from *abcr*. Most reagents were used without additional purification, except when mentioned explicitly in the procedure. Solvents were kept dry before use with molecular sieves (0.4 nm).

S2.1.3. Amino acid protection

In a 100 mL wide-neck bottle, amino acid (25 mmol), and KOH (25 mmol) are dissolved in water (30 mL) on an ice bath. To a near-room-temperature, vigorously stirred solution, di-*tert*-butyl dicarbonate (DIBOC, 26 mmol) in ethanol (15 mL) is added dropwise. A rapid evolution of CO_2 ensues, and when it subsides (after about 20-30 minutes), another portion of KOH (25 mmol) is added. The reaction is left, stirred, to finish overnight and afterwards evaporated under reduced pressure to remove ethanol. The solution is diluted with water (20 mL), transferred to a 250 mL beaker and placed on an ice bath. First portion of HCl (25 mmol) can be added dropwise, reasonably fast, as a 10-15% solution, and afterwards the pH of the solution is carefully brought to ~2.5 (not less than 2.2) with a 2 M HCl solution. Precipitation may occur; if so, 50 mL of ethyl acetate may be added to immediately dissolve the free carboxylic acid. The solution or mixture is then moved to a separatory funnel and extracted at least once with 50 mL and thrice with 25 mL of ethyl acetate. The combined organic phase is back-extracted with pure water, brine, dried over anhydrous sodium sulphate and evaporated under reduced pressure to yield 86-98% of pure BOC-protected amino acid**.**

S2.1.4. Diamide synthesis

N-BOC amino acid (12.5 mmol) is placed in a 50 mL wide-neck bottle with a screw-on cap (flushed with argon) and dissolved in 20 mL of dry dichloromethane. N,N'-Dicyclohexylcarbodiimide (12.5 mmol) is added, bottle closed, and the mixture stirred until full dissolution (precipitation or clouding may occur with some amino acids). 1,3-Bis-(3-aminopropyl)-tetramethyldisiloxane (5 mmol) is then added dropwise to the stirred reaction mixture, preferably temporarily placed on an ice bath. The reaction vessel is closed again and stirred for 48 hours. The white precipitate of dicyclohexylurea is then filtered out, and the solution evaporated under reduced pressure in a round-bottom flask. The resulting oil is dissolved in diethyl ether (~10 mL) and a varying amount of petroleum ether is added, depending on the lipophilicity of the amino acid (1 mL for glycine, 3 mL for β-alanine, 5 mL for L-proline or GABA). No permanent clouding should occur at this point, and diethyl ether can be added if it does. The solution is left for 48 hours at 4°C and if any precipitate or oil forms, it is discarded. Glycine and β-alanine derivatives can be obtained by addition of petroleum ether (10 and 20 mL respectively), to the remaining solution, with gentle mixing. The separating oil is left at ~4°C to gather at the bottom and harden, the supernatant exchanged for a less polar solvent mixture (10 mL, proportionally half the amount of diethyl ether used before) and the product dispersed on an ultrasonic bath, then left to gather at the bottom of the flask again. After the supernatant is again removed and the oil evaporated under reduced pressure, the pure BOC-protected diamide is obtained in a 42-68% yield. The less polar amino acid derivatives can be obtained similarly, but the original diethyl ether solution is first evaporated, the oil mixed with 1-2 mL of fresh diethyl ether and then with 20-30 mL of petroleum ether; the mixture is left at -15°C overnight. The resulting oil is treated as described above, but washed with pure petroleum ether.
Note: BOC-protected diamides of more polar amino acids (e.g. asparagine) can also be isolated by this method, by using THF-diethyl ether mixture instead of diethyl ether-petroleum ether mixture to separate the fraction of intermediate polarity.

S2.1.5. Diamide deprotection

Deprotection proceeds smoothly, both with anhydrous methanolic HCl solution and TMS-Cl (trimethylsilyl chloride). The solution (5 mmol of diamide, 10 mL of MeOH, 20 mmol of HCl / TMSCl) is stirred at room temperature overnight, then evaporated under reduced pressure, redissolved in anhydrous ethanol and again evaporated, then lyophilized.
Note: any solid precipitate forming in the methanolic solution stems from diamide impurities and can be safely discarded.

S2.1.6. Bis(guanidine) synthesis

A 50 mL round-bottom flask with deprotected diamide dichloride salt (5 mmol), 1*H*-pyrazole-1-carboxamidine hydrochloride (10 mmol; note: the reagent should be decolorized beforehand if the commercial sample isn't white), triethylamine (TEA, 11 mmol) and acetonitrile (15 mL) is equipped with a reflux condenser. The mixture is heated to reflux with vigorous stirring and the reaction is carried out for 90 minutes, during which a viscous, yellowish oil of bis-guanidine dichloride forms on the bottom of the flask. The solution is allowed to stand and cool to ~40°C (not less), and the supernatant is removed before TEA hydrochloride begins to precipitate. Fresh acetonitrile (8 mL) is added and the mixture brought to reflux, with vigorous stirring, again. After cooling, the supernatant is removed and added to the first one. Final yield of the bis-guanidine (slightly yellow oil or waxy semisolid) after lyophilization varies from 36-88%, with less polar amino acid derivatives giving lower yields due to their solubility in warm acetonitrile. If the yield is not satisfactory, the organic phase can be evaporated under reduced pressure, the residue redissolved in 2-propanol and refrigerated overnight. Most of the TEA hydrochloride precipitates and can be filtered out. The 2-propanol is then evaporated under reduced pressure and the resulting oil refluxed in chloroform, then again cooled overnight. The supernatant is discarded and the procedure repeated to give an additional fraction (up to 34% for L-proline derivative, total 70%) of pure bis-guanidine.

S2.2. Compound characterization

GLY

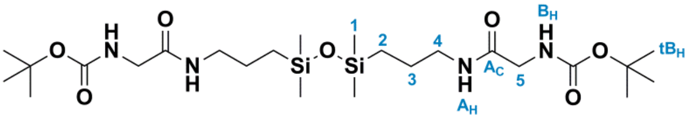

1A

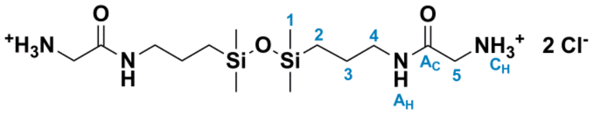

1B

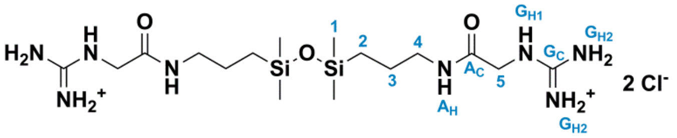

1C

βALA

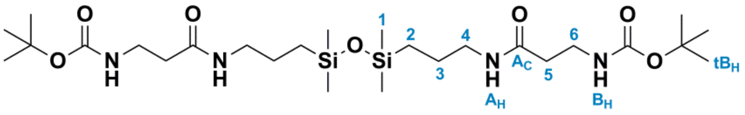

2A

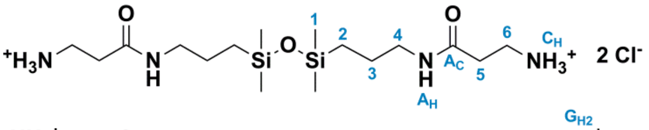

2B

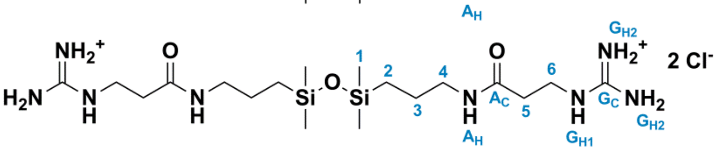

2C

GABA

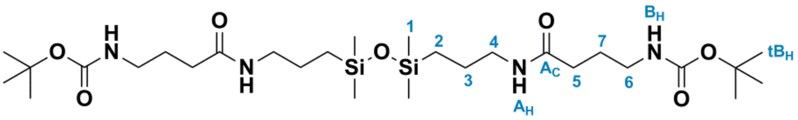

3A

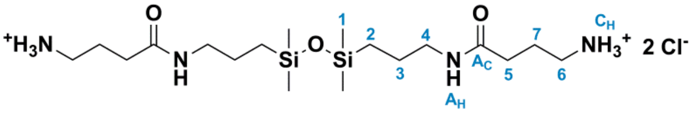

3B

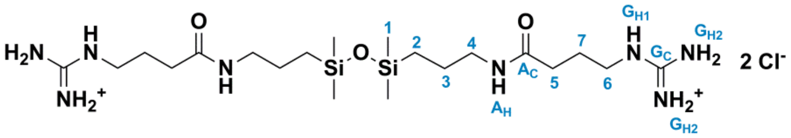

3C

PRO

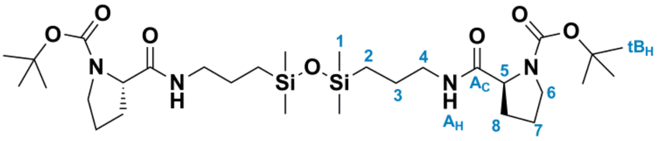

4A

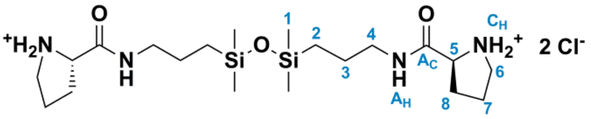

4B

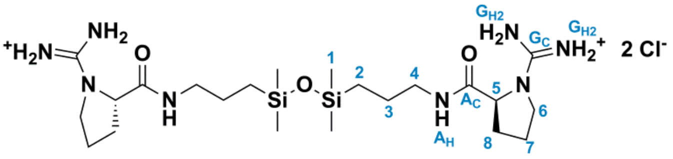

4C

Signal chemical shift [ppm], multiplicity, coupling constant												
^{1}H	1A	1B	1C	2A	2B	2C	3A	3B	3C	4A	4B	4C
1	0.045; s	0.051; s	0.049; s	0.051; s	0.047; s	0.047; s	0.048; s	0.047; s	0.045; s	0.038; s	0.049; s	0.046; s
2	0.50; m	0.50; m	0.49; m	0.50; m	0.48; m	0.48; m	0.51; m	0.47; m	0.47; m	0.49; m	0.49; m	0.48; m
3	1.53; m	1.44; m	1.43; m	1.52; m	1.41; m	1.41; m	1.53; m	1.40; m	1.40; m	1.49; m	1.44; m	1.42; m
4	3.23; q, *J*=6.8 Hz	3.09; q, *J*=6.8 Hz	3.06; q, *J*=6.5 Hz	3.21; dt, *J*=7.2, 6.1 Hz	2.96; q, *J*=6.5 Hz	3.03; q, *J*=6.4 Hz	3.21; dt, *J*=7.1, 6.1 Hz	3.01; q, *J*=6.8 Hz	3.10; q, *J*=6.7 Hz	3.19; br[a]	3.10; m	3.04; m
5	3.80; d, *J*=5.6 Hz	3.53; q, *J*=5.5 Hz	3.85; d (br), *J*=4.0 Hz	2.42; t, *J*-6.1 Hz	2.50; t, *J*=7.2 Hz	2.36; t, *J*=6.6 Hz	2.23; t, *J*=7.1 Hz	2.21; t, *J*=7.2 Hz	2.15; t, *J*=7.3 Hz	4.25; br[a]	4.15; p, *J*=6.8 Hz	4.58, dd, *J*=8.0, 1.3 Hz
6	-	-	-	3.40; q, *J*=6.2 Hz	3.03; q, *J*=6.5 Hz	3.34; q, *J*=6.5 Hz	3.17; q, *J*=6.4 Hz	2.76; m	3.02; q, *J*=6.8 Hz	3.46; br[a]	3.20; m	3.37, 3.53; m
7	-	-	-	-	-	-	1.81; p, *J*=6.8 Hz	1.80; p, *J*=7.2 Hz	1.68; p, *J*=7.2 Hz	1.87; br[a]	1.88; p, *J*=6.8 Hz	1.94; m
8	-	-	-	-	-	-	-	-	-	1.87, 2.13; br	1.80, 2.31; m	1.92, 2.16; m
A_H	6.76; br	8.64; t, *J*=5.5 Hz	8.31; t, *J*=5.5 Hz	6.42; br	8.22; t, *J*=5.5 Hz	8.21; t, *J*=5.5 Hz	6.71; br	8.12; t, *J*=5.2 Hz	8.04; t, *J*=5.6 Hz	6.29; 7.01, br	8.78; t, *J*=5.5 Hz	8.35; t, *J*=5.5 Hz
B_H	5.67; br	-	-	5.32; br	-	-	5.04; br	-	-	-	-	-
tB_H	1.45; s	-	-	1.43; s	-	-	1.44; s	-	-	1.46; s	-	-
C_H	-	8.25; br	-	-	8.04; br	-	-	8.20; br	-	-	8.50, 10.17 br	-
G_{H1}	-	-	7.75; br	-	-	7.73; t, *J*=5.5 Hz		-	7.91; t, *J*=5.6 Hz	-	-	-
G_{H2}	-	-	7.0-7.8; br	-	-	6.8-7.5, 7.4-7.9; br	-	-	6.8-7.4, 7.3-7.8; br	-	-	7.4-7.6; br

Table S2.2.1. ^{1}H NMR data**:** all disiloxane diamides (**A**-**C**).

Spectra (400 Mhz) of **A** were obtained in $CDCl_3$ (0.05 M solutions), and of **B** and **C** in $(CD_3)_2SO$ (0.05 M solutions); TMS was used as internal standard (0.00 ppm). [a] Broadening of signals from ^{1}H nuclei far from the siloxane bridge is most probably caused by the hindered movement of proline residues (due to bulky BOC protecting groups).

Signal chemical shift [ppm]										
^{13}C	1	2	3	4	5	6	7	8	A_C	G_C
**1C**	0.4	15.2	23.1	41.8	43.5	-	-	-	167.0	157.6
**2C**	0.4	15.2	23.1	41.7	34.7	37.3	-	-	167.0	157.2
**3C**	0.4	15.2	23.2	41.7	32.0	40.2	24.8	-	171.5	157.1
**4C**	0.4	15.1	22.9	41.8	60.0	47.8	23.0	30.9	169.6	155.1

Table S2.2.2. ^{13}C NMR data**:** disiloxane bis(guanidines) (**C**).

All spectra (100 Mhz) were obtained in $(CD_3)_2SO$ (0.05 M solutions); due to low visibility of TMS, residual solvent peak from DMSO (39.53 ppm)[2] was used as reference. [a] Broadening of signals further from the siloxane bridge (due to hindered rotation of proline residues, caused by bulky BOC groups) prevents the resolution of most signals into well-defined multiplets.

Element	H [%]		C [%]		N [%]	
	Calculated	Found	Calculated	Found	Calculated	Found
**1C**	7.76	7.88	36.98	36.88	21.56	21.32
**2C**	8.10	8.16	39.47	39.40	20.46	20.33
**3C**	8.40	8.48	41.72	41.59	19.46	19.29
**4C**	8.07	8.17	44.06	43.92	18.68	18.38

Table S2.2.3. Elemental analysis data: disiloxane bis(guanidines) (**C**).

S3. Salt metatheses and solubility studies

S3.1. Materials

All salts were obtained from *Merck* and were of analytical or higher grade. Compounds prone to moisture absorption (or unstable hydrates) were dried in appropriate temperatures and low pressure to constant mass. Bis-guanidines **1-4C** were additionally lyophilized once more before use.

S3.2 Estimation of aqueous solubilities / K_{sp} of salts

Determination of solubility of different bis-guanidinium salts was meant as a purely comparative study, as we were more interested in the dynamic organization of matter and not the standardized thermodynamic solubility values. The product of molar concentrations at the moment of phase separation was an attractive and not overcomplicated way to compare different ion pairings. The experiments were therefore performed without further corrections (e.g. for ion activities), as follows:

To a stirred vial containing 1 mL of bis-guanidine dihydrochloride **1-4C** solution (0.1 M, aqueous) and 0.5 mL of water, aliquots of a given salt solution (1 N, i.e. 1 M for monosodium salts, and 0.5 M for disodium salts, aqueous), were added, at constant temperature (25°C). The permanent clouding / precipitation point was detected easily by visual observation. The standard aliquot was 25 µL, with the exception of dichromate. To differentiate between the very low solubilities of bis-guanidine chromates, the additions were consecutively: 10 µL, 10 µL, 15 µL, 15 µL, after which standard 25 µL doses followed. The additions were continued, with a standard 30 second delay between them, until a total volume of 1 mL was achieved (2.5 mL total solution volume, a 5-fold counterion molar excess). The K_{sp} was calculated for two ion concentrations: corresponding to the aliquot resulting in clouding or precipitation, and the one before. The obtained ranges (presented in the main paper in **Figure 1**) served as an uncorrected estimation of aqueous solubility. All experiments were repeated twice (with new solutions), and gave exactly the same results.

S3.3. Preparation of different bis(guanidinium) salts

Salt metatheses was performed on the basis of different solubility of corresponding sodium and bis-guanidinium salts. Salts of monovalent anions were obtained either in **S3.2** or, if they did not separate in those conditions, by 24 h equilibration of the dichloride salt solution with a 10-fold excess of a suitable sodium salt in ethanol. In the former case, they were purified by washing with ice-cold water, dissolution in absolute ethanol and low-pressure evaporation (repeated twice), and final dissolution in ethanol/THF/diethyl ether mixture of the lowest possible polarity. This solution was filtered to remove the residual inorganic salts, evaporated under reduced pressure, and lyophilized. In the latter case (thiocyanates, nitrites, **βALA-NO$_3$**) the mixture was filtered, evaporated, dissolved in a less polar solvent and filtered again; this was repeated until no more solid precipitate formed. The product was lyophilized. Salts of divalent anions were purified following **S3.2** by washing with ice-cold water (usually twice), and repeated dispersion (ultrasonic bath) in absolute ethanol, followed by evaporation and lyophilization. Substitution of chlorides with another counterion was tested each time using a solution of $AgNO_3$. Most salts that separated in **S3.2** as a liquid phase did not crystallize or solidify during or after purification. Only salts of **GUA** were predominantly solid in pure form. This allowed for a comparative evaluation of their melting points:

GUA salt	Benzoate	Nitrate	Nitrite	Chromate	Molybdate	Sulphate
Melting point [°C]	146-148	156-159	159-161	~200 (dec.)	~225 (dec.)	~275 (dec.)

Table S3.3.1. Comparison of melting points: **GUA** salts.

Although lower melting points / decomposition temperatures coincide with lower aqueous solubilities of salts (in a given - monovalent / divalent counterion - category), the correlation is tentative.

S4. Additional crystallographic data

S4.1. Additional figures

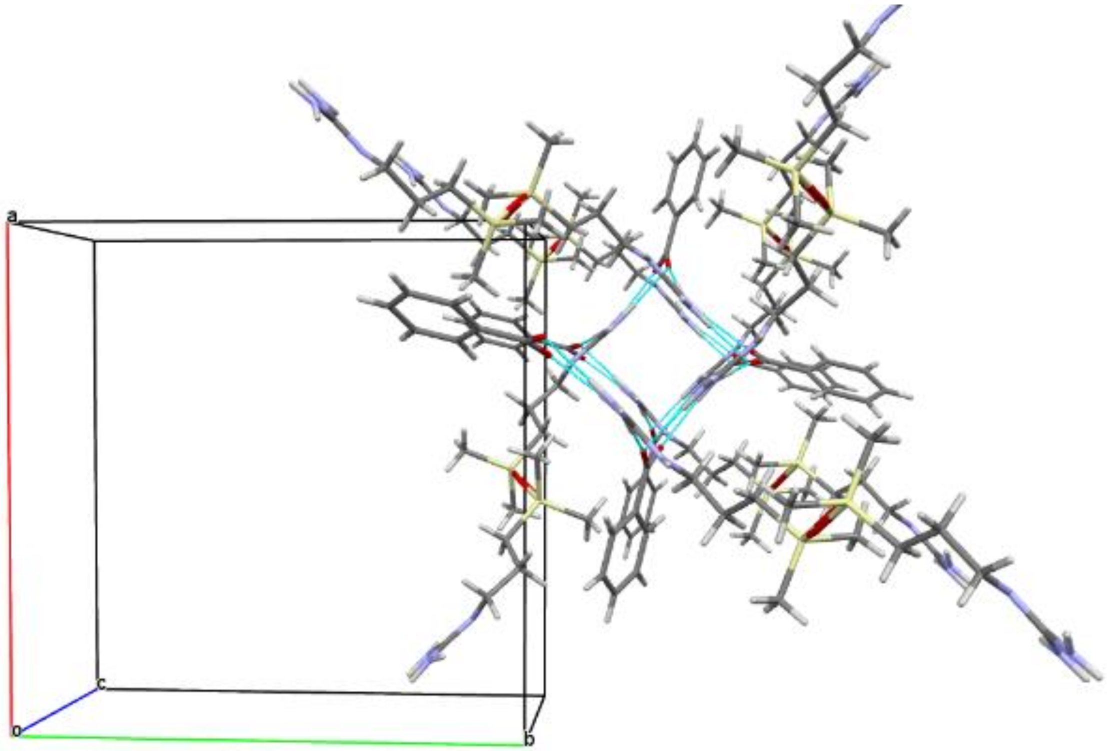

Figure S3.1.1 The NH---O bonded helix fragment of the **GUA-Bz** structure viewed down [z].

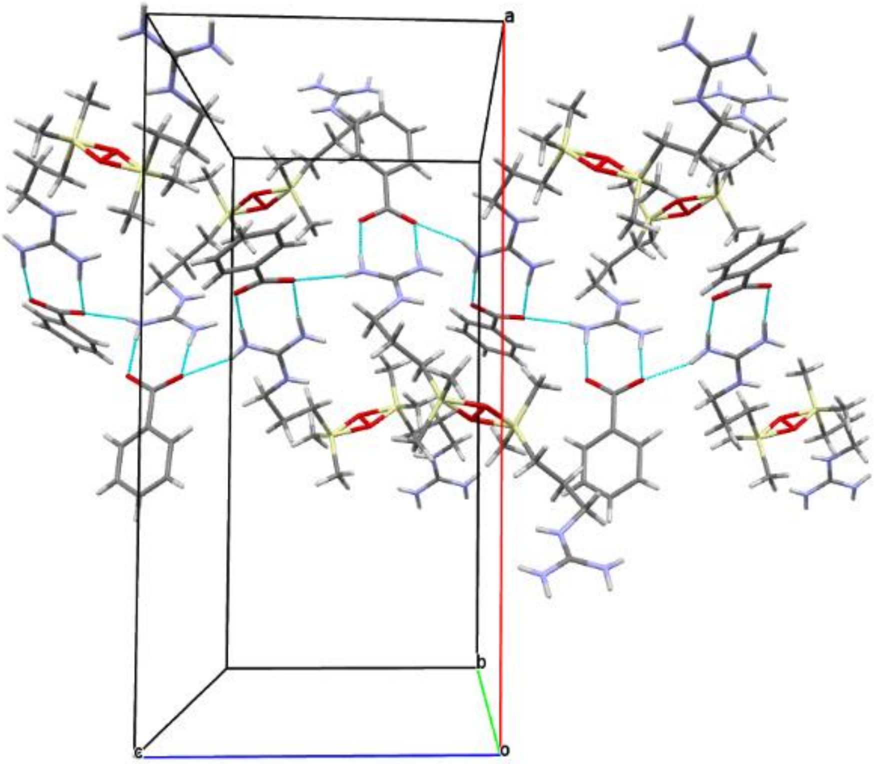

Figure S4.1.2. The NH---O bonded helix fragment of the **GUA-Bz** structure viewed down [y].

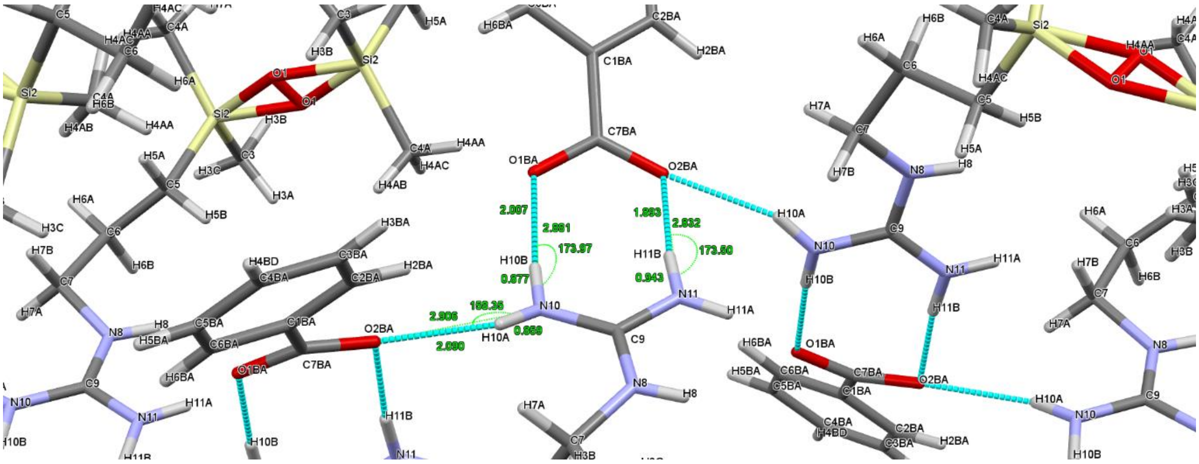

Figure S4.1.3. Close-up fragment of the NH---O bonded helix in **GUA-Bz** structure with denoted hydrogen bond angles and distances (cyan dotted lines).

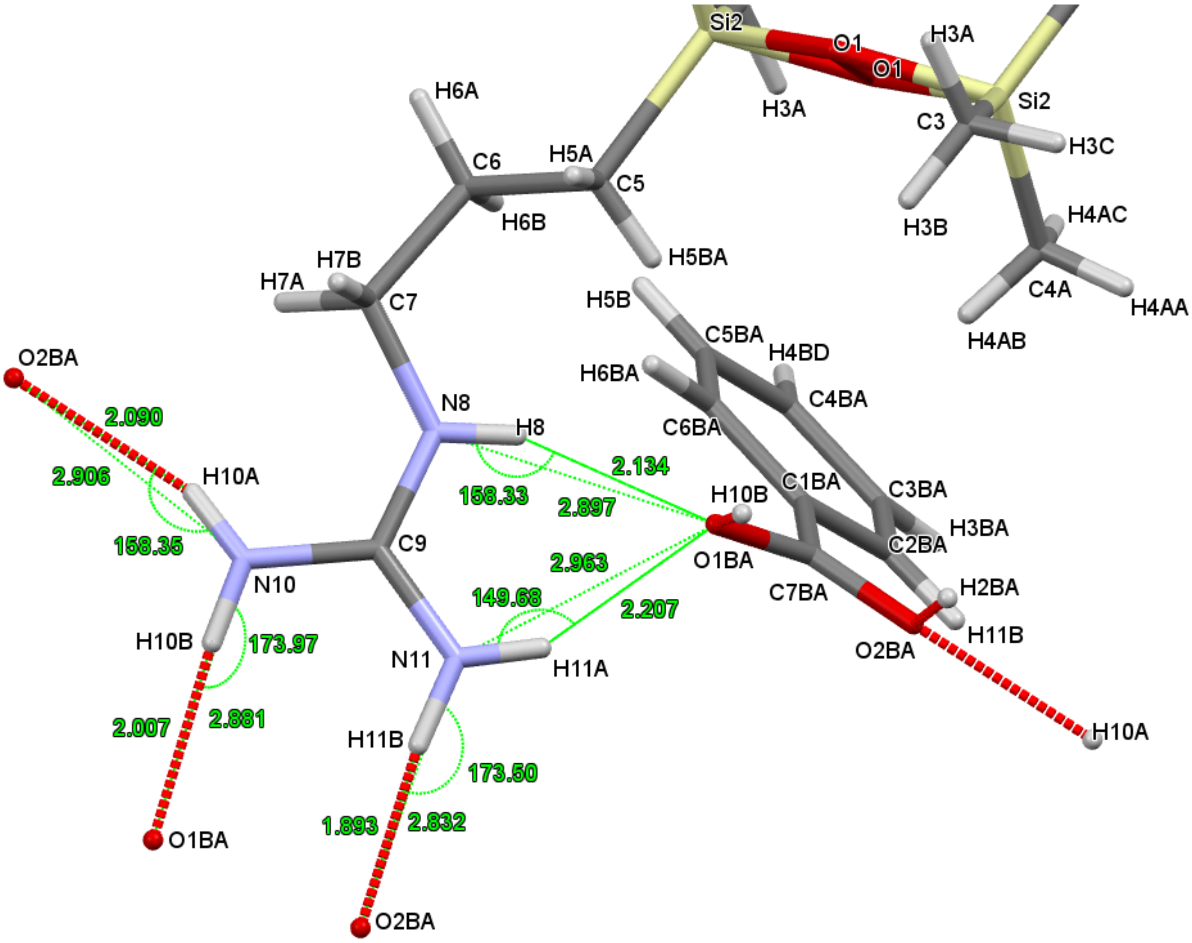

Figure S4.1.4. All hydrogen bonds formed by the guanidinium moiety in **GUA-Bz** structure, with denoted bond angles and distances (green and red dotted lines).

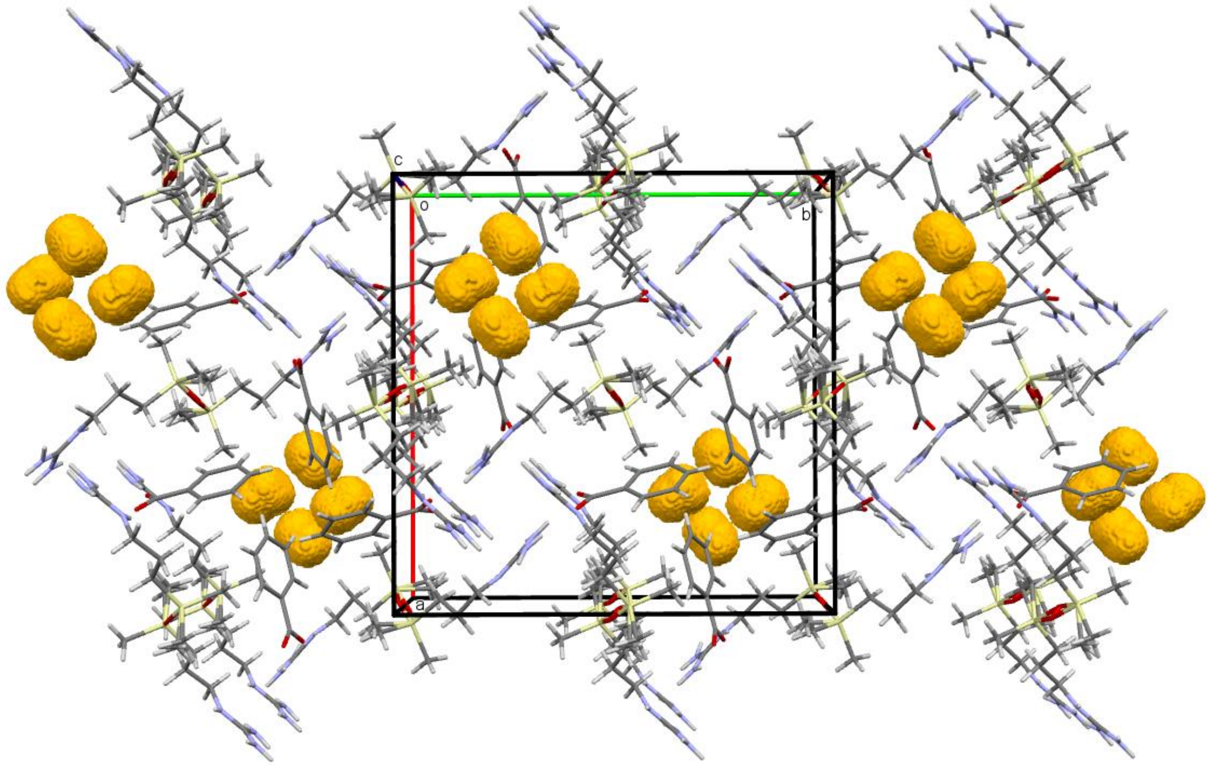

Figure S4.1.5. The **GUA-Bz** structure viewed along the [z] axis. The void regions probed with spheres 1.0 Å in 0.1 Å steps are indicated in gold. This drawing can be viewed in 3-dimensions as an autostereogram.[3]

S5. Spectra glossary

S5.1 NMR spectra

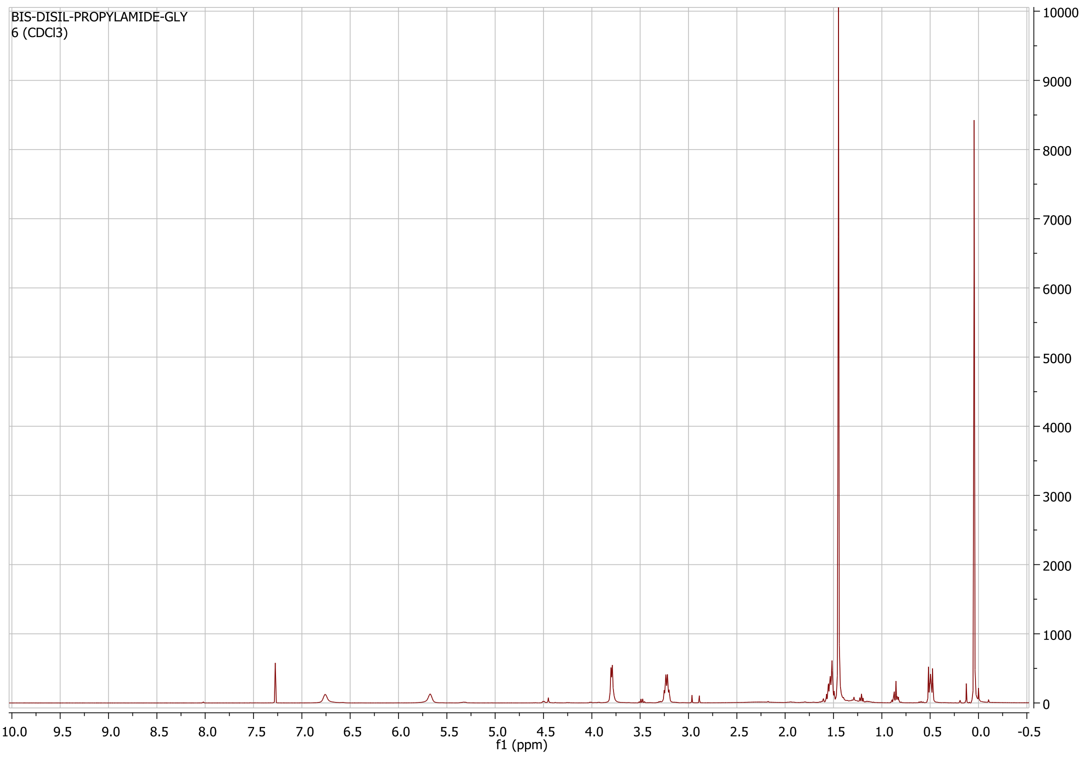

Figure S5.1.1. ^{1}H NMR spectrum of compound **1A**.

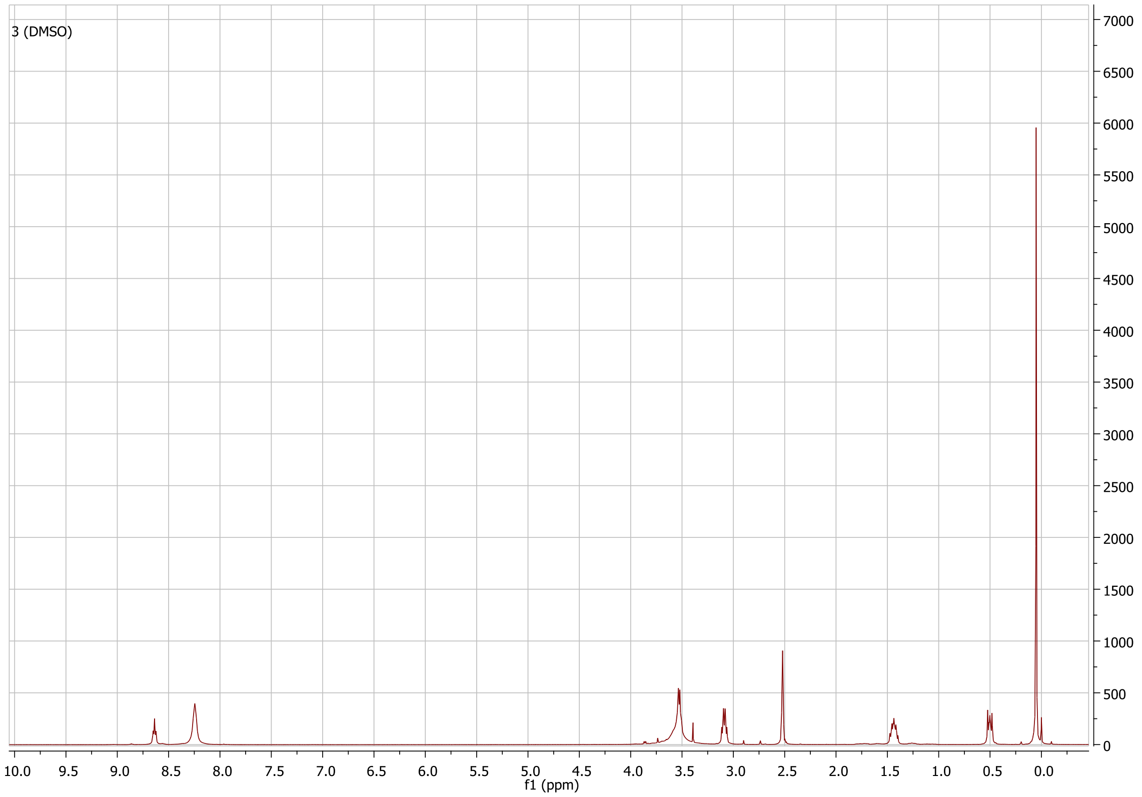

Figure S5.1.2. ^{1}H NMR spectrum of compound **1B**.

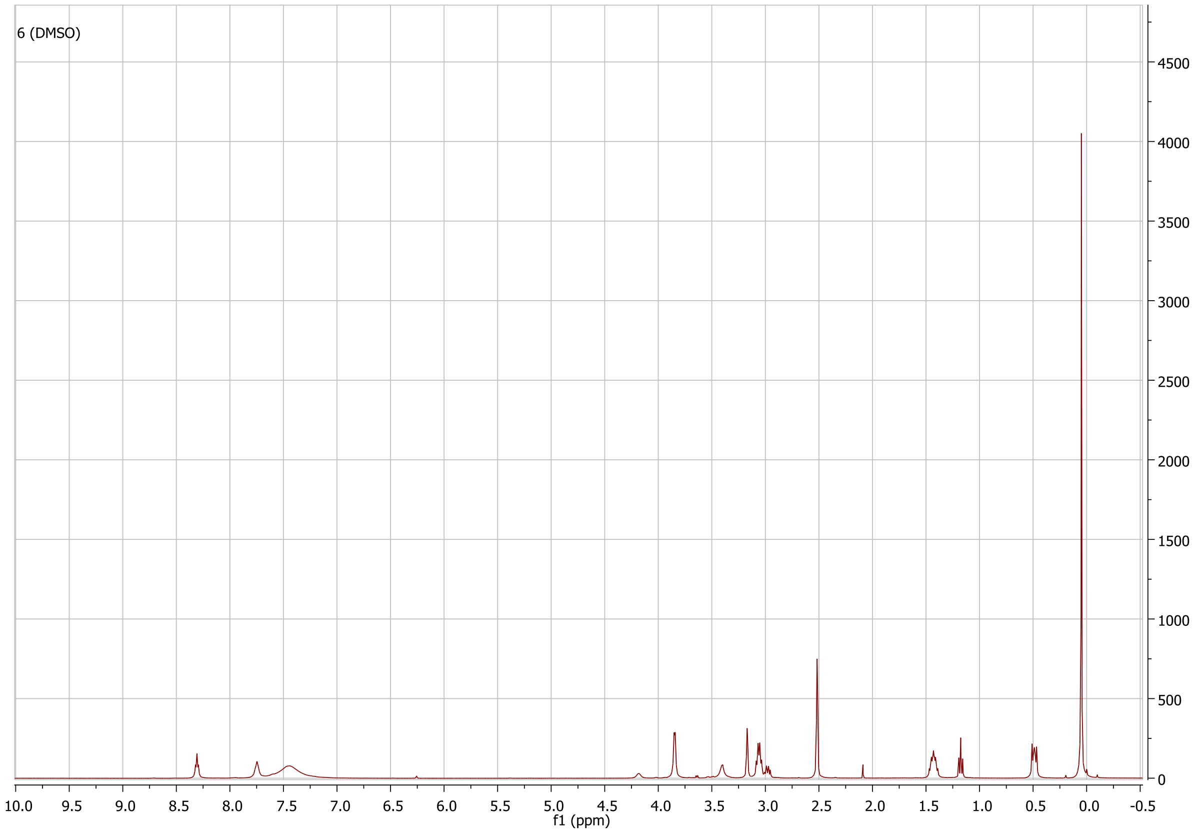

Figure S5.1.3. ^{1}H NMR spectrum of compound **1C** (**GLY**).

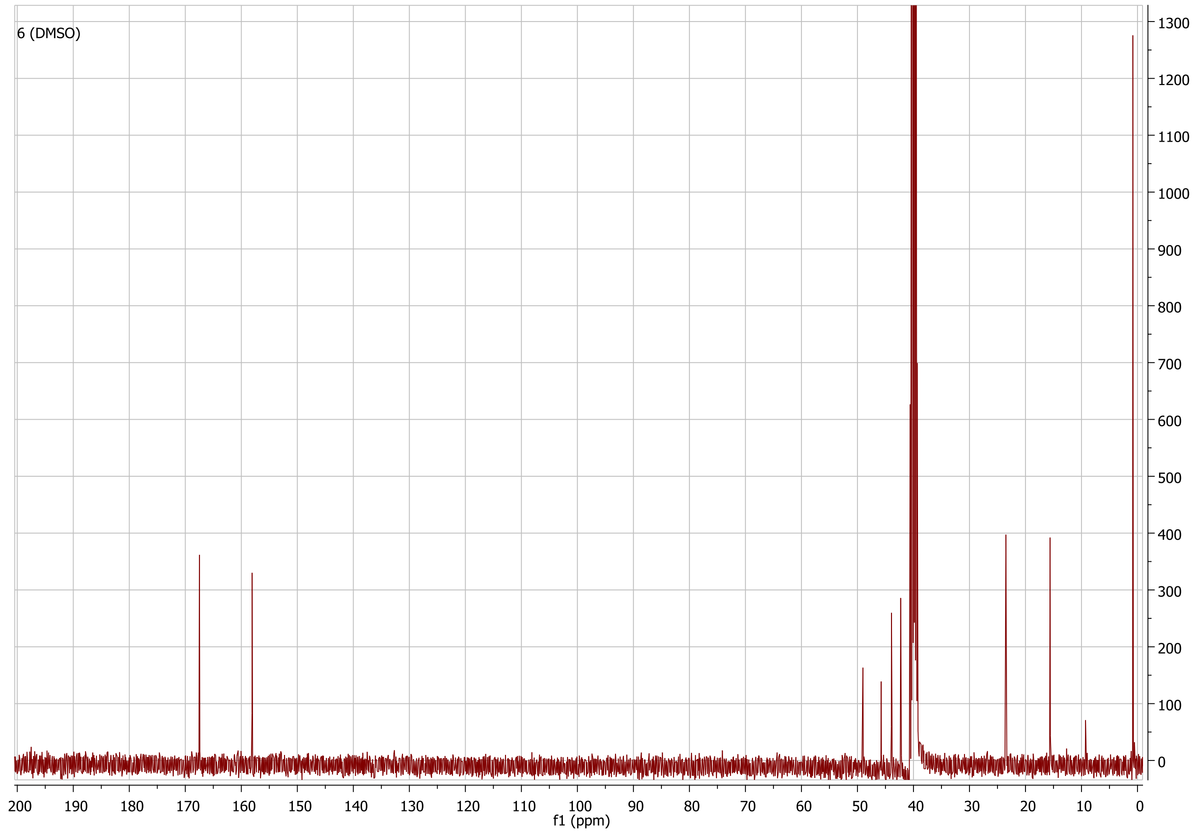

Figure S5.1.4. ^{13}C NMR spectrum of compound **1C** (**GLY**).

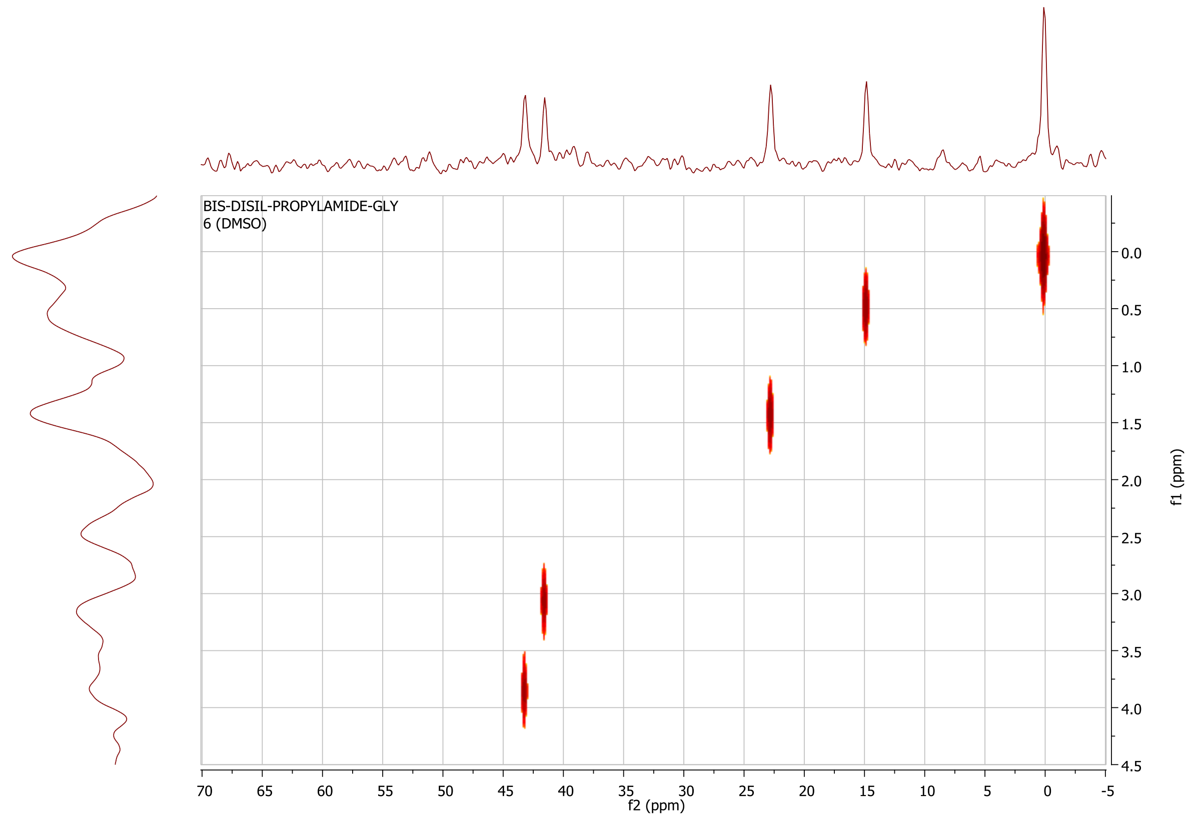

Figure S5.1.5. 2D spectrum (^{1}H-^{13}C HETCOR) of compound **1C** (**GLY**).

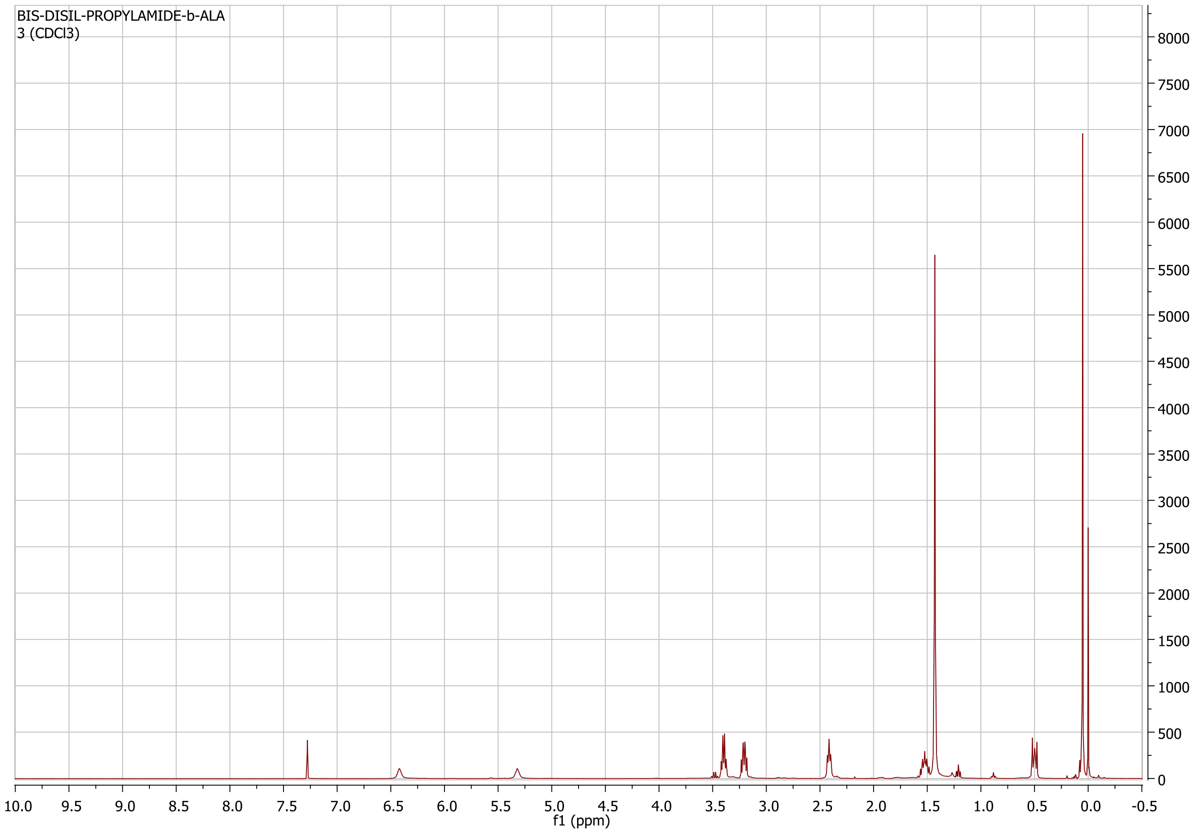

Figure S5.1.6. ^{1}H NMR spectrum of compound **2A**.

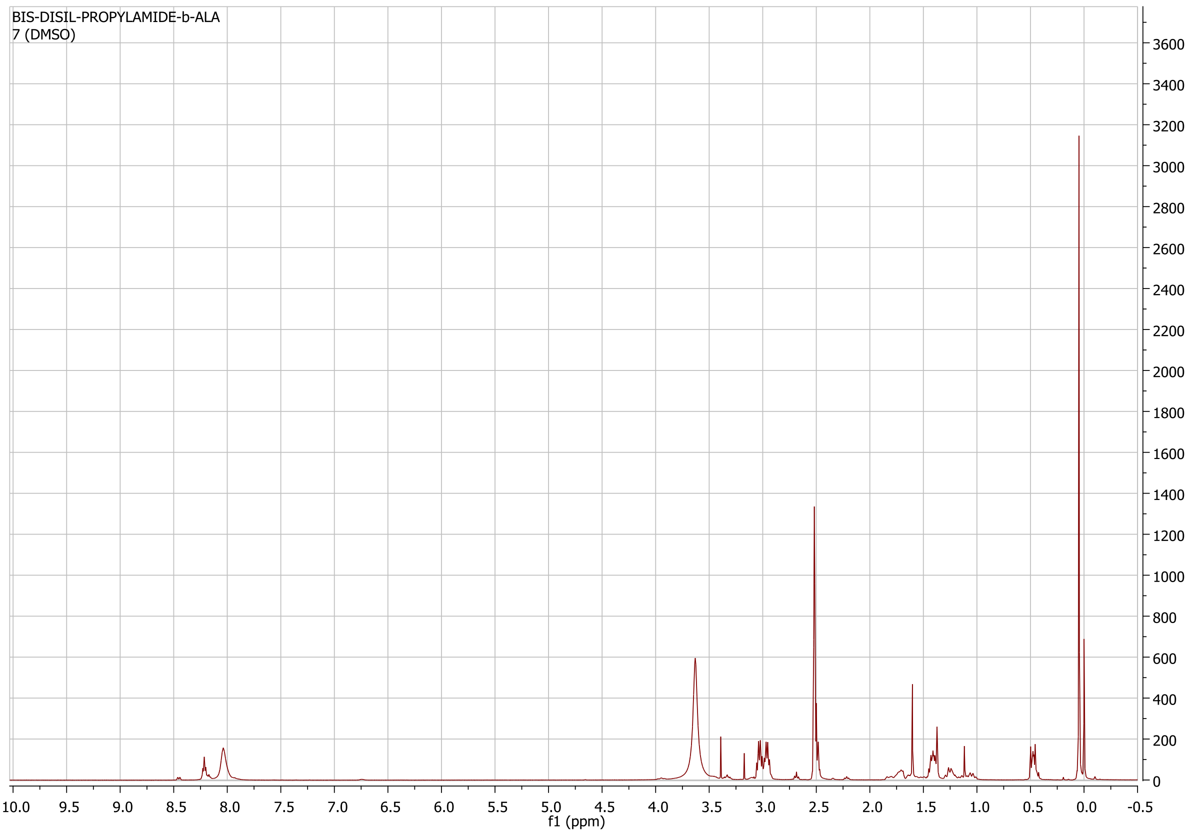

Figure S5.1.7. ^{1}H NMR spectrum of compound **2B**.

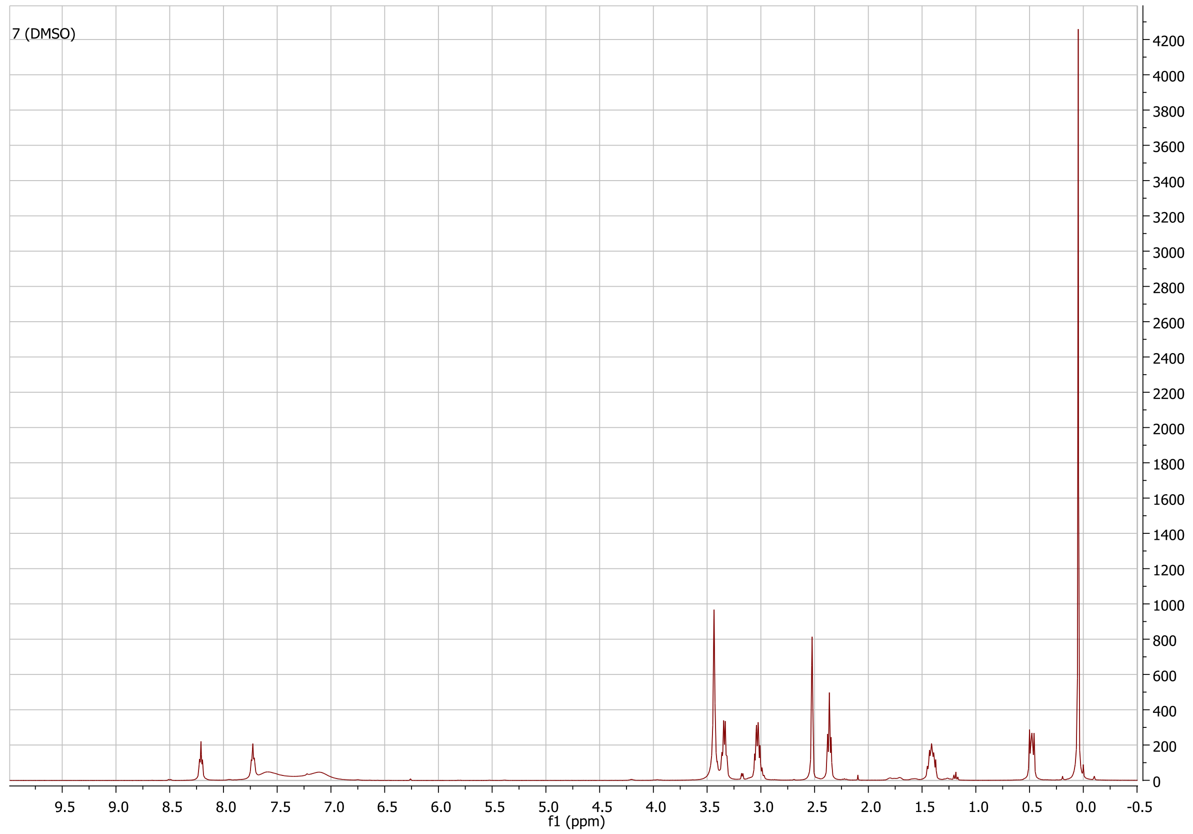

Figure S5.1.8. ^{1}H NMR spectrum of compound **2C** (**βALA**).

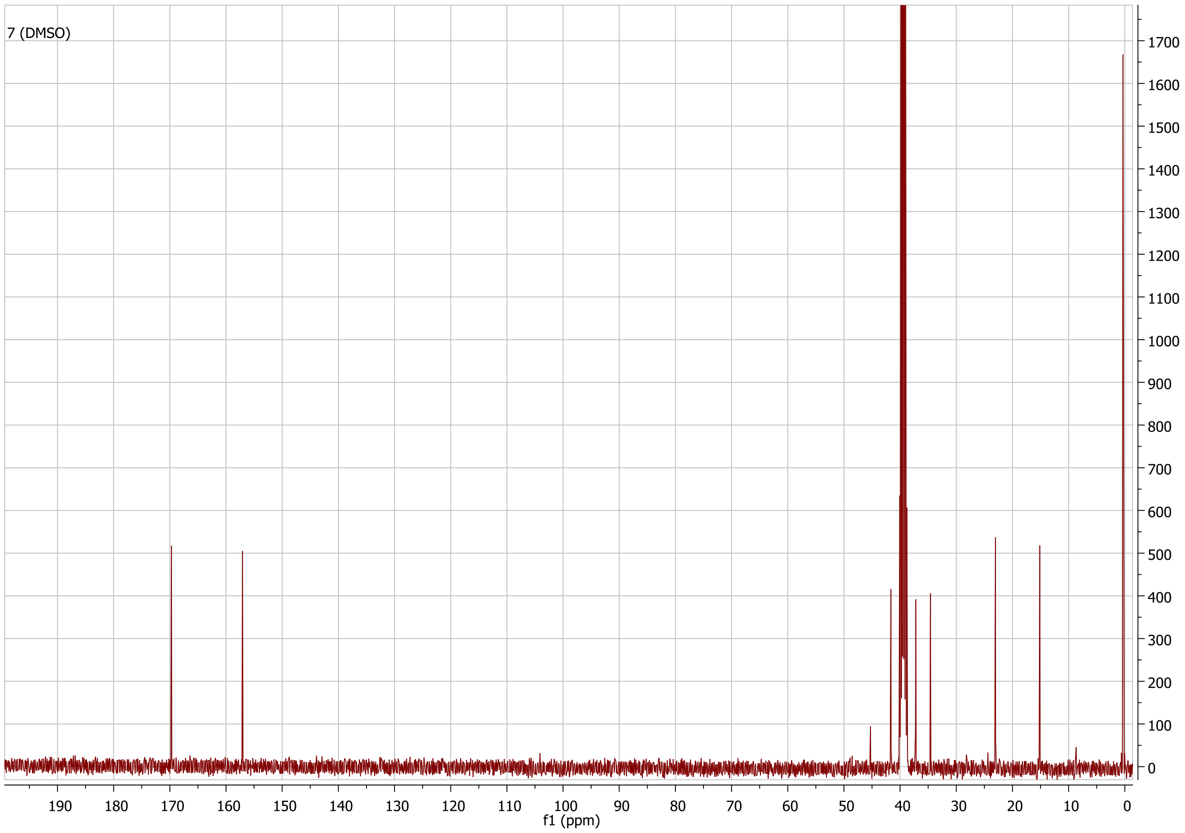

Figure S5.1.9. ^{13}C NMR spectrum of compound **2C** (**βALA**).

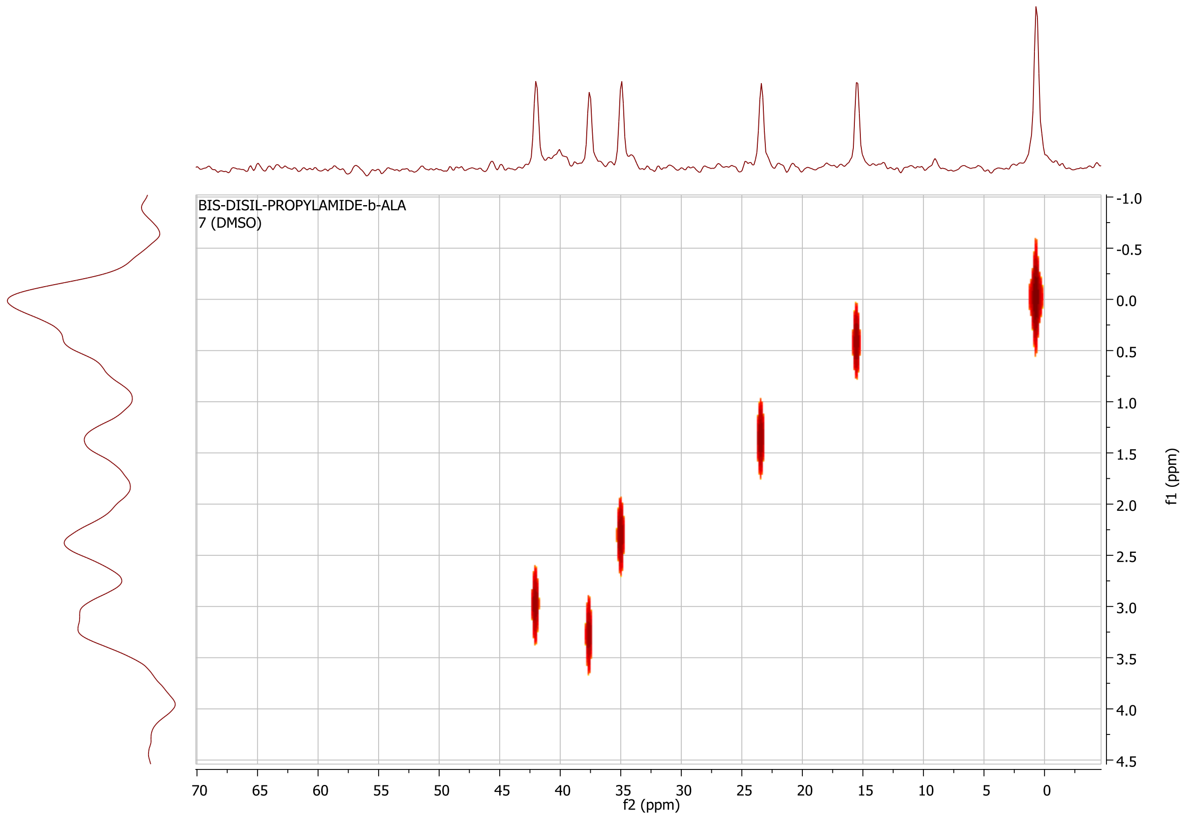

Figure S5.1.10. 2D spectrum (^{1}H-^{13}C HETCOR) of compound **2C** (**βALA**).

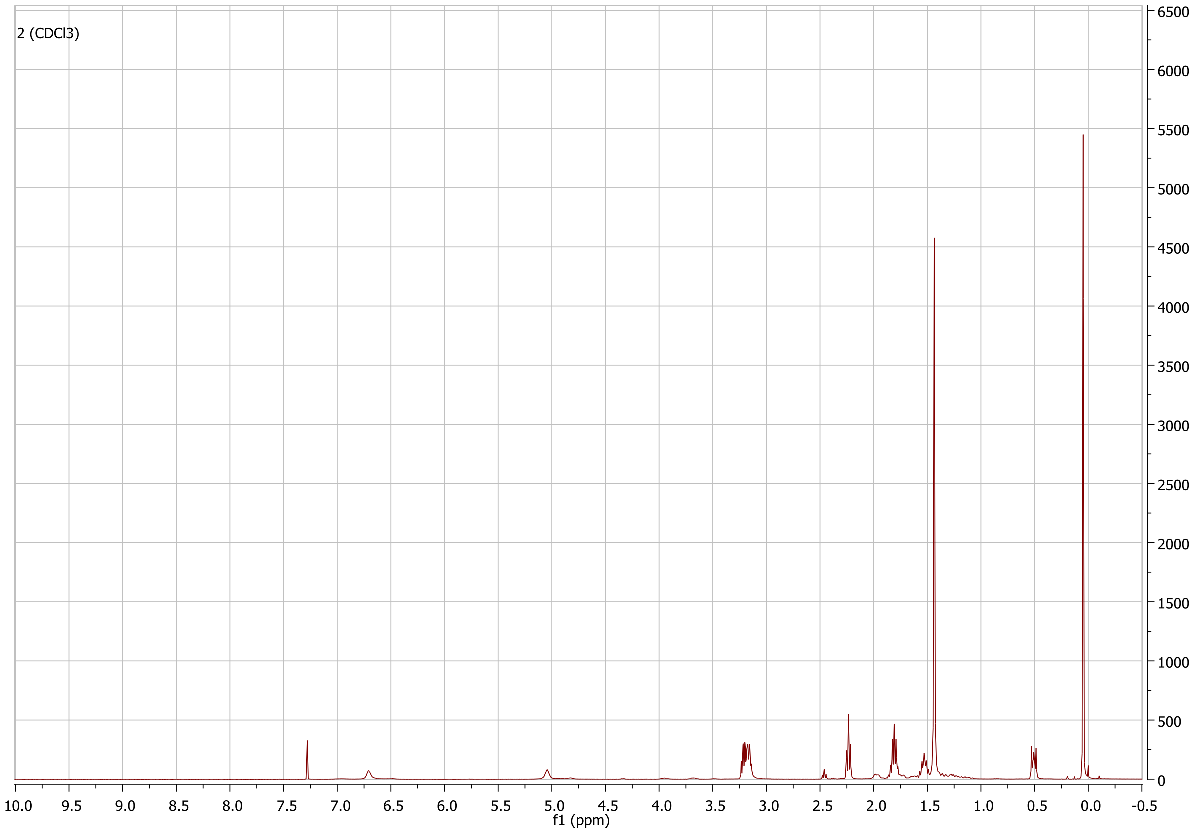

Figure S5.1.11. ^{1}H NMR spectrum of compound **3A**.

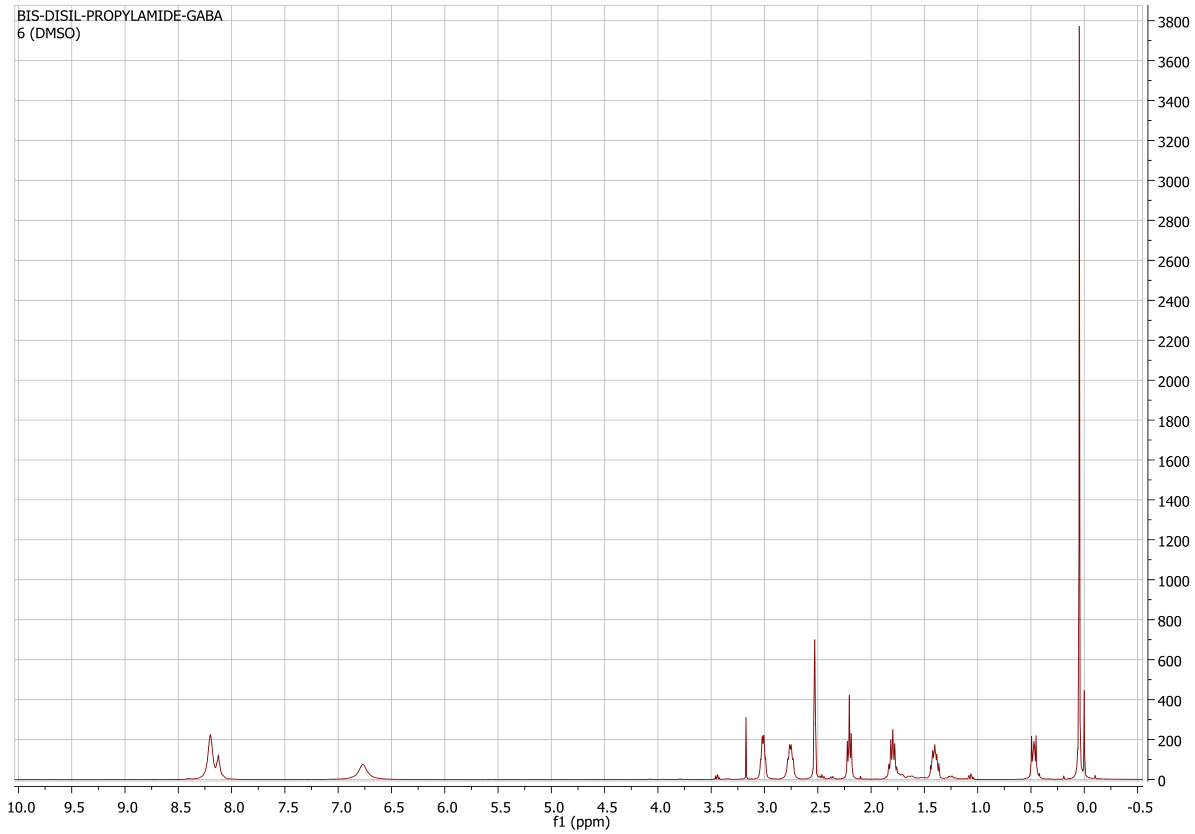

Figure S5.1.12. ^{1}H NMR spectrum of compound **3B**.

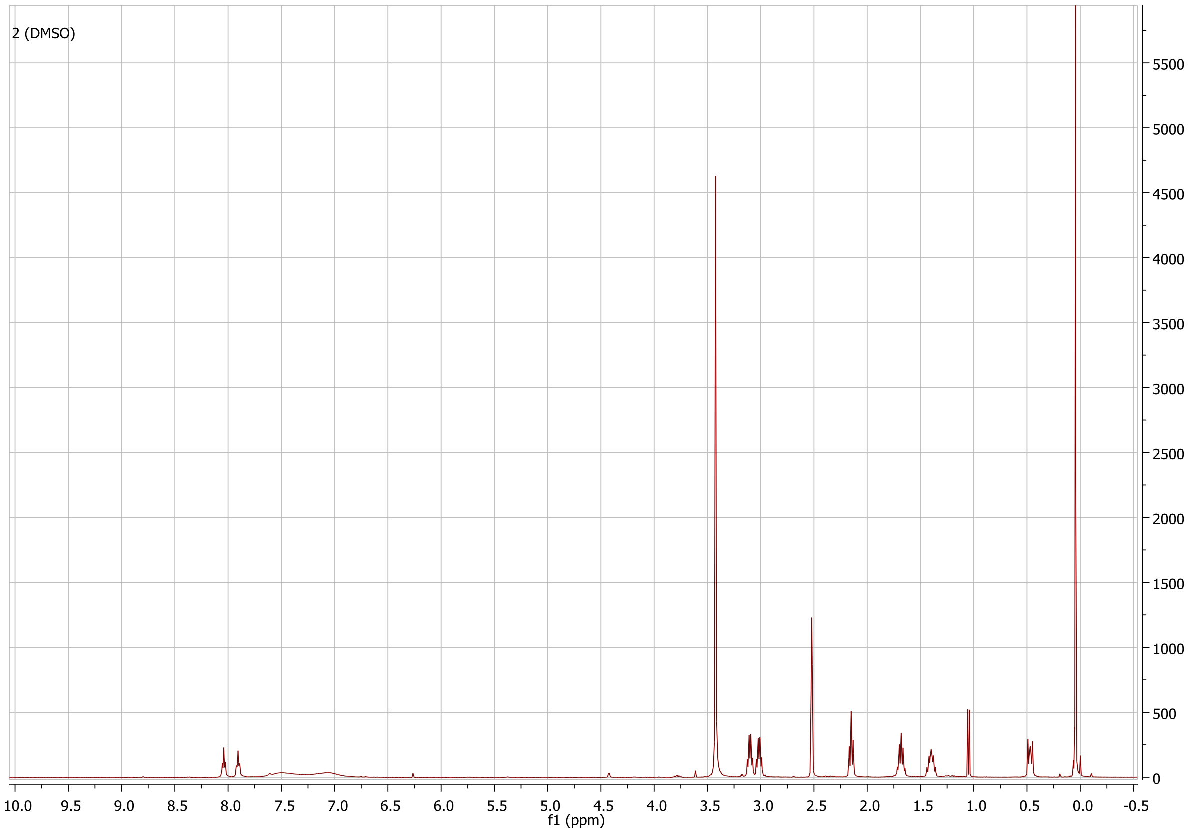

Figure S5.1.13. ^{1}H NMR spectrum of compound **3C** (**GABA**).

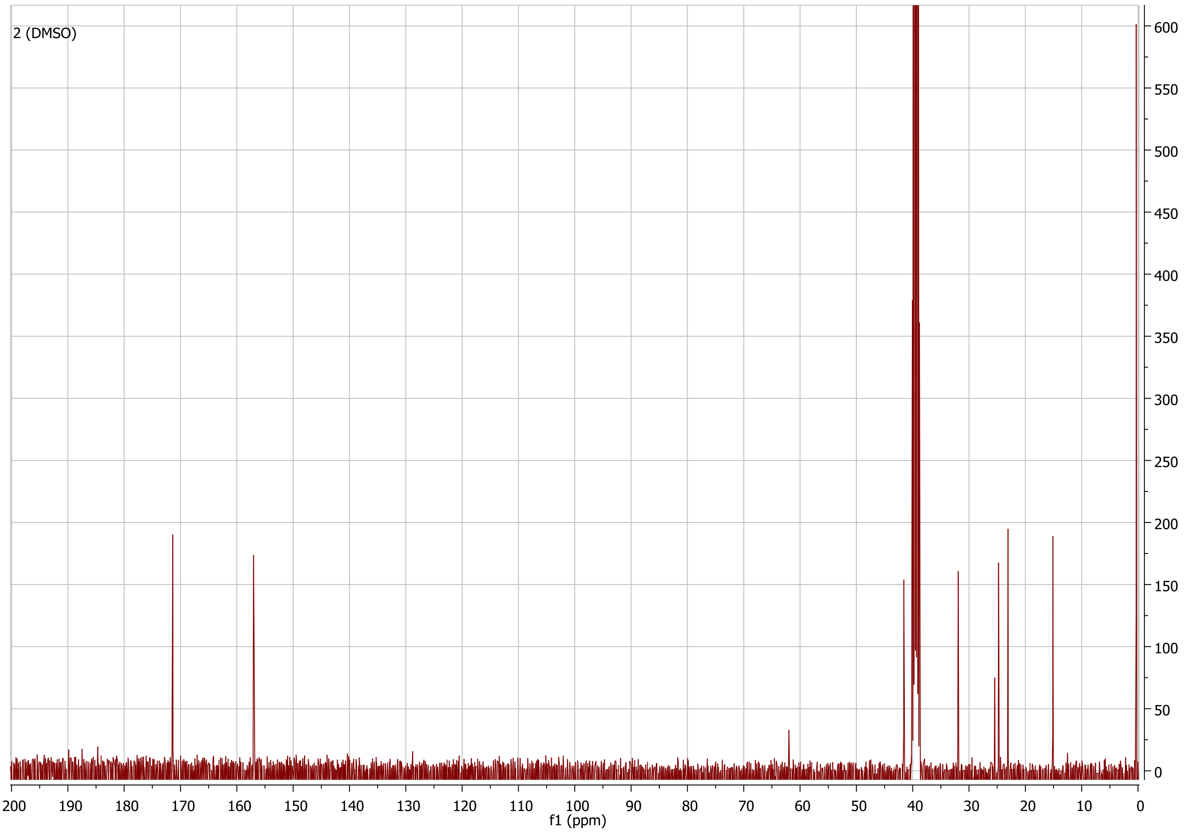

Figure S5.1.14. ^{13}C NMR spectrum of compound **3C** (**GABA**).

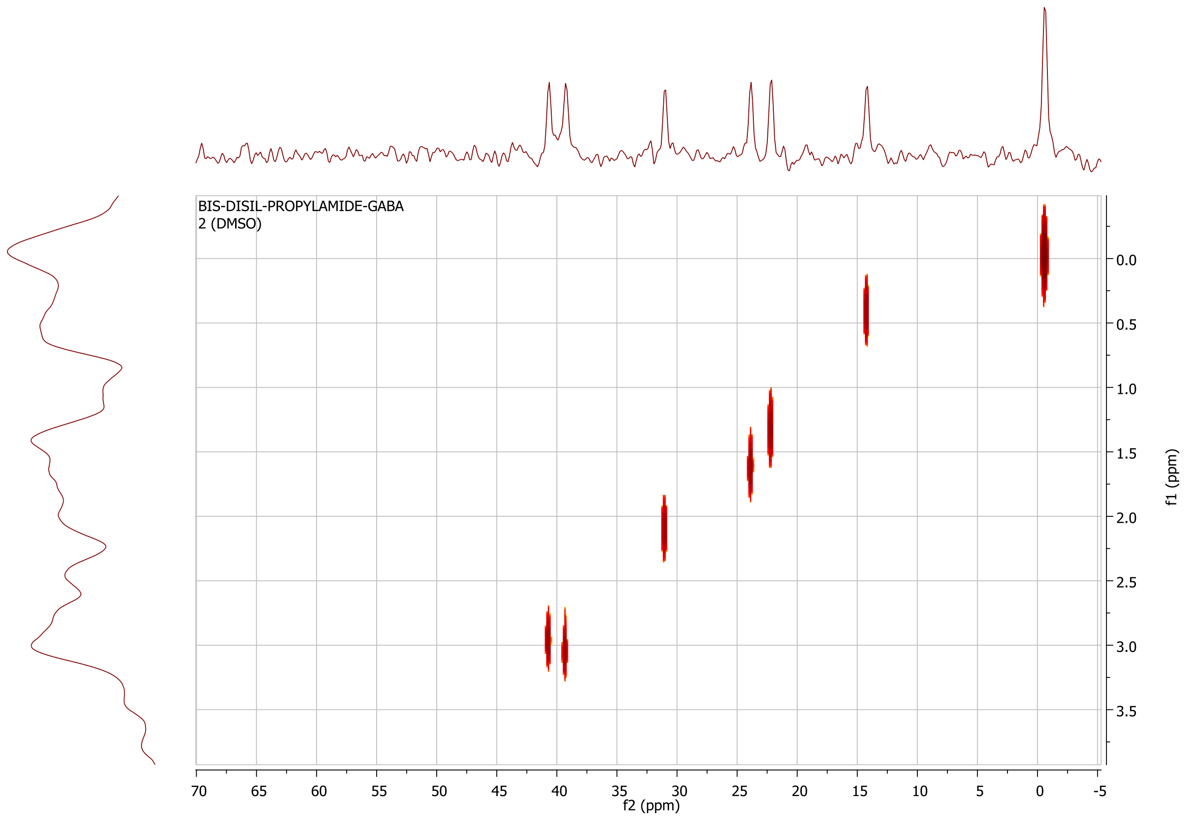

Figure S5.1.15. 2D spectrum (^1H-^{13}C HETCOR) of compound **3C** (**GABA**).

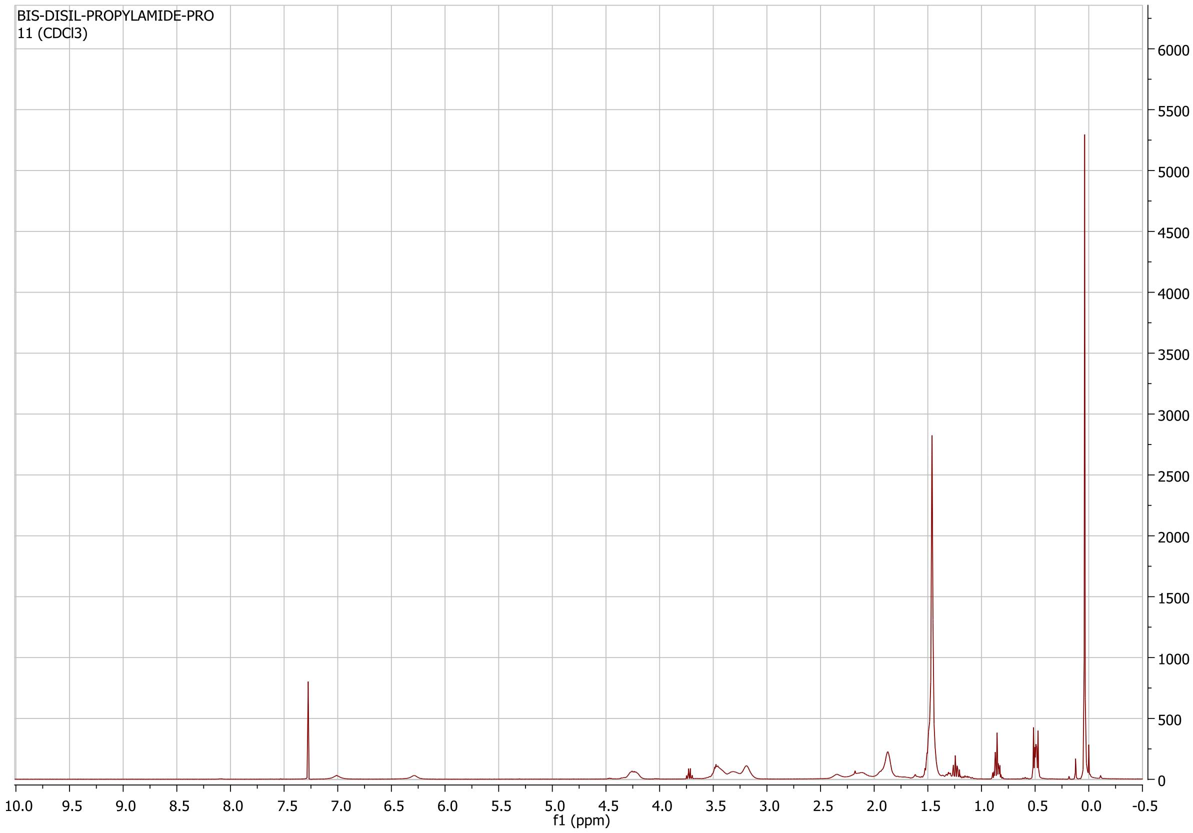

Figure S5.1.16. ^1H NMR spectrum of compound **4A.**

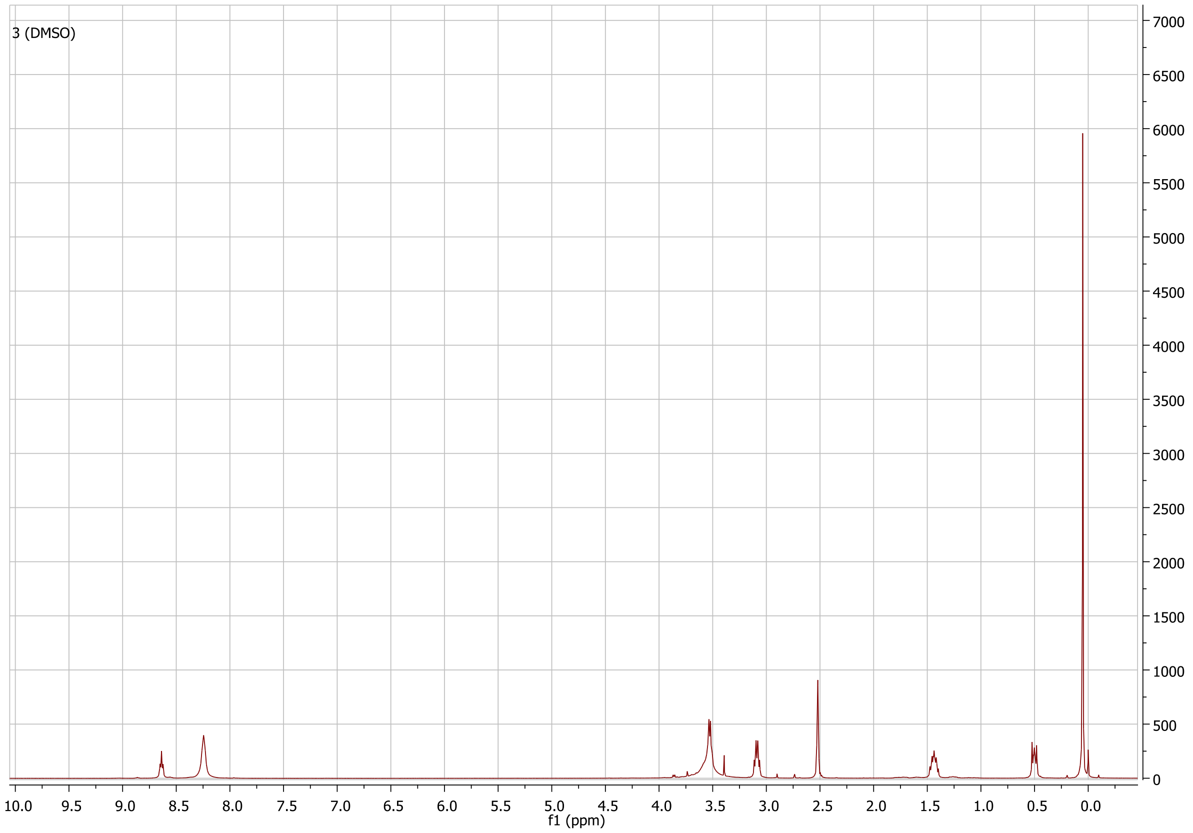

Figure S5.1.17. ^{1}H NMR spectrum of compound **4B**.

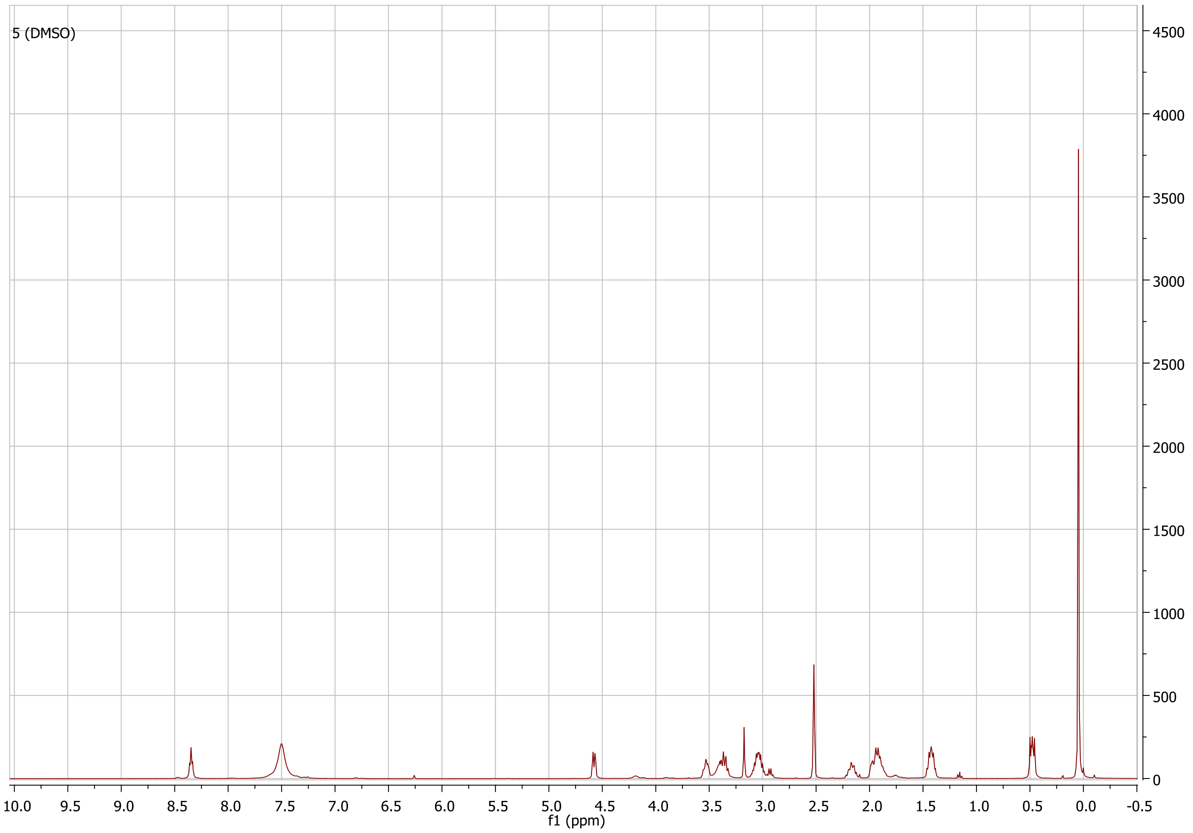

Figure S5.1.18. ^{1}H NMR spectrum of compound **4C** (**PRO**).

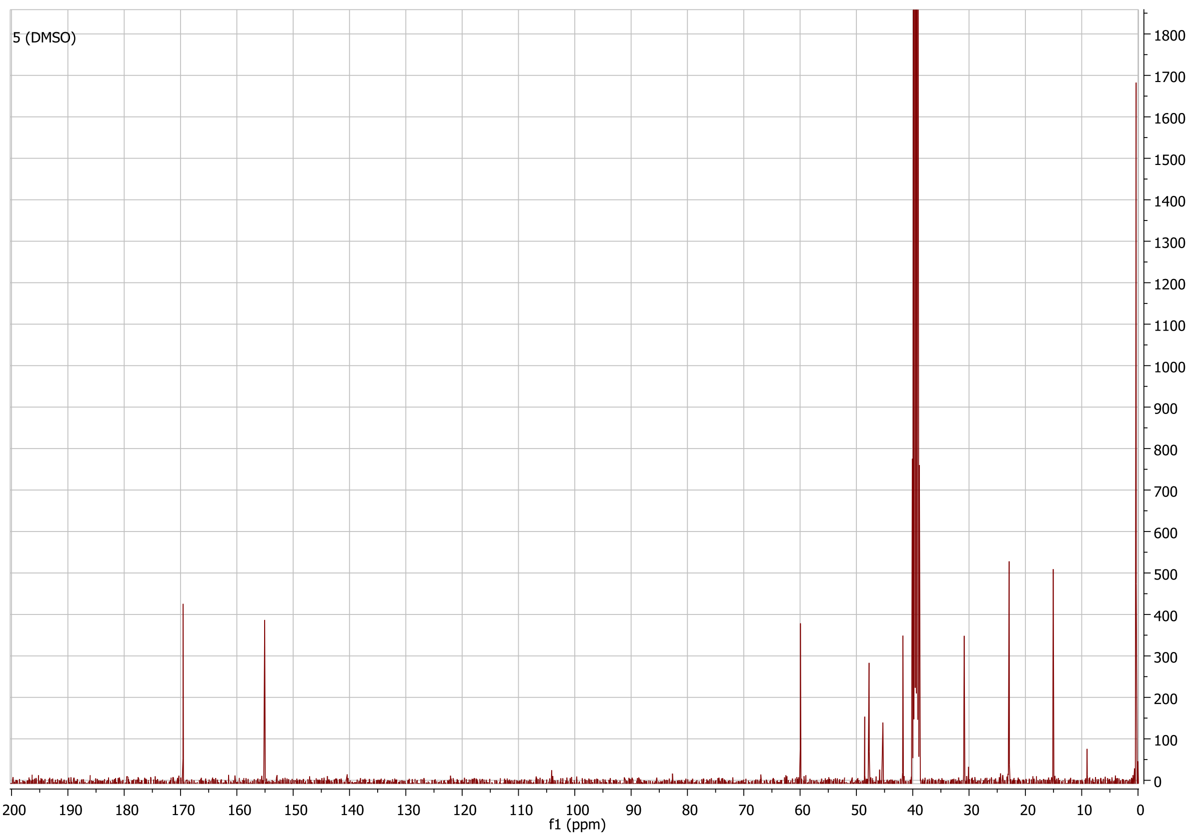

Figure S5.1.19. ^{13}C NMR spectrum of compound **4C** (**PRO**).

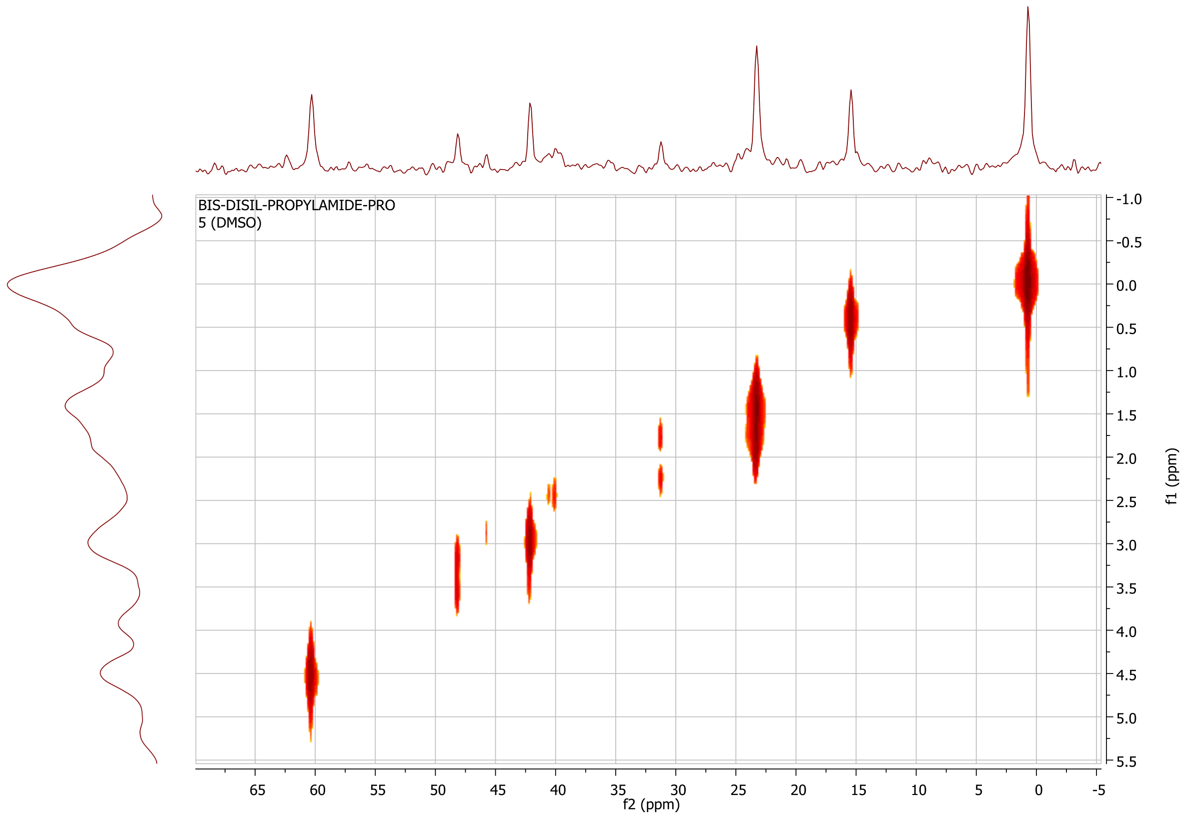

Figure S5.1.20. 2D spectrum (^{1}H-^{13}C HETCOR) of compound **4C** (**PRO**).

S5.2 FT-IR spectra

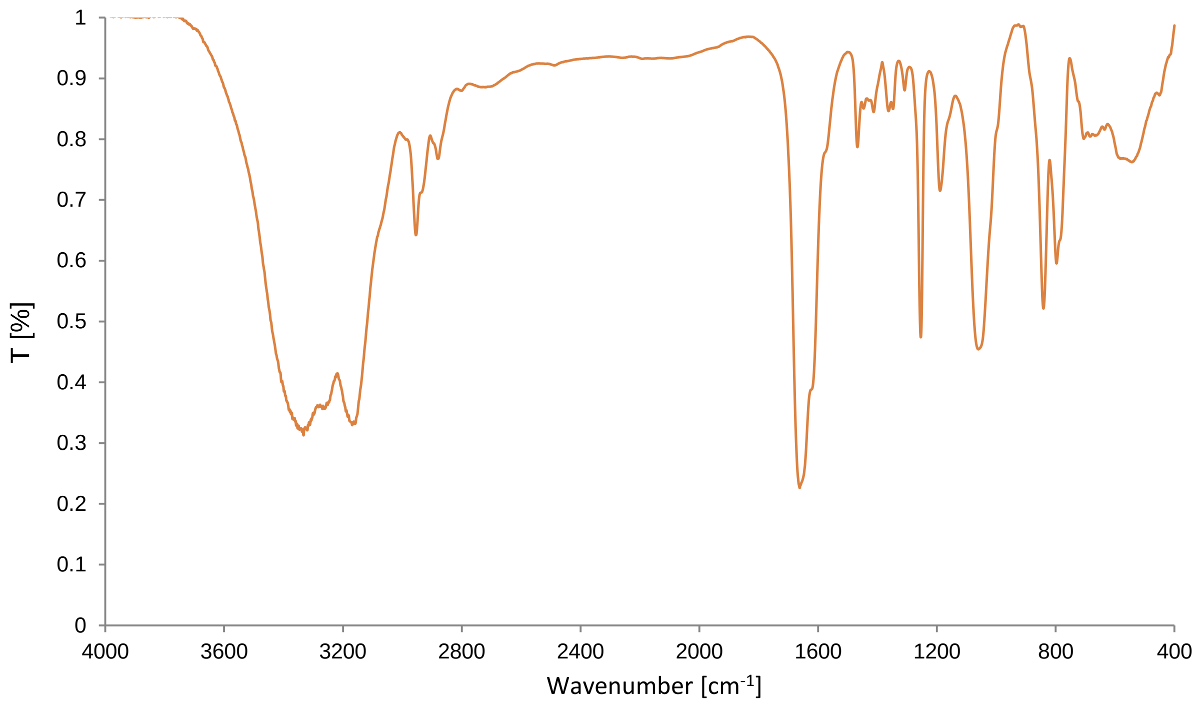

Figure S5.2.1. FT-IR spectrum of 1,3-bis(3-guanidinopropyl)-tetramethyldisiloxane dichloride (**GUA**).

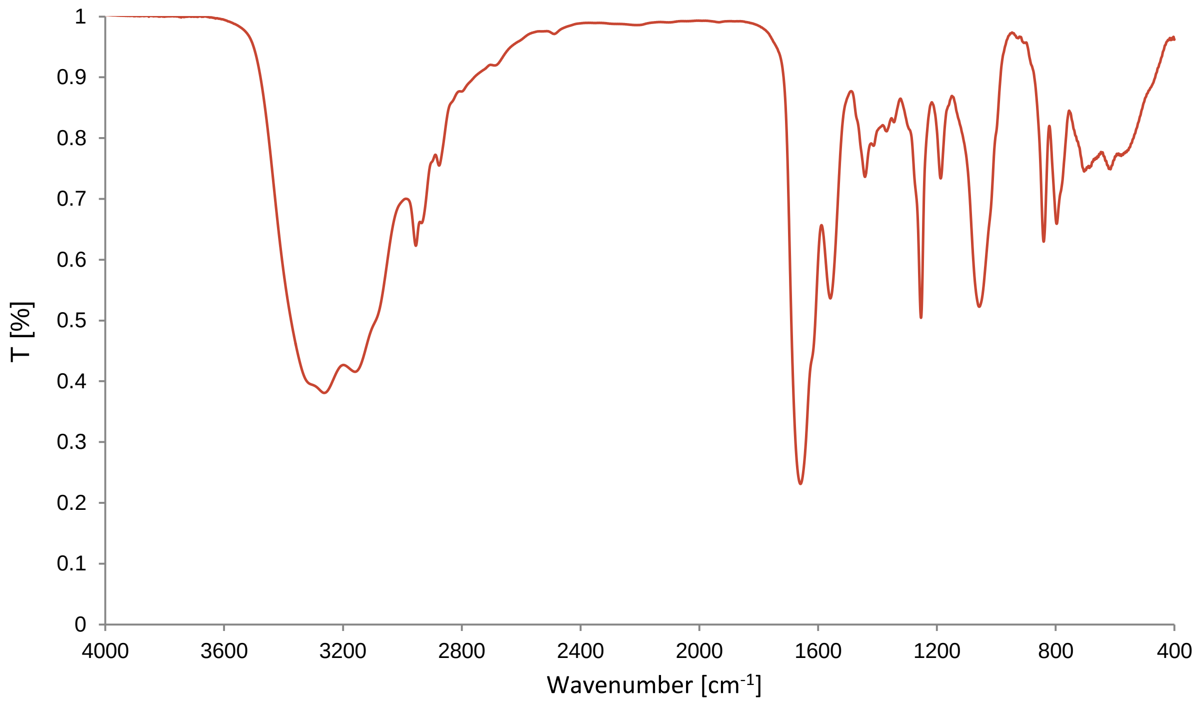

Figure S5.2.2. FT-IR spectrum of **1C** (**GLY**).

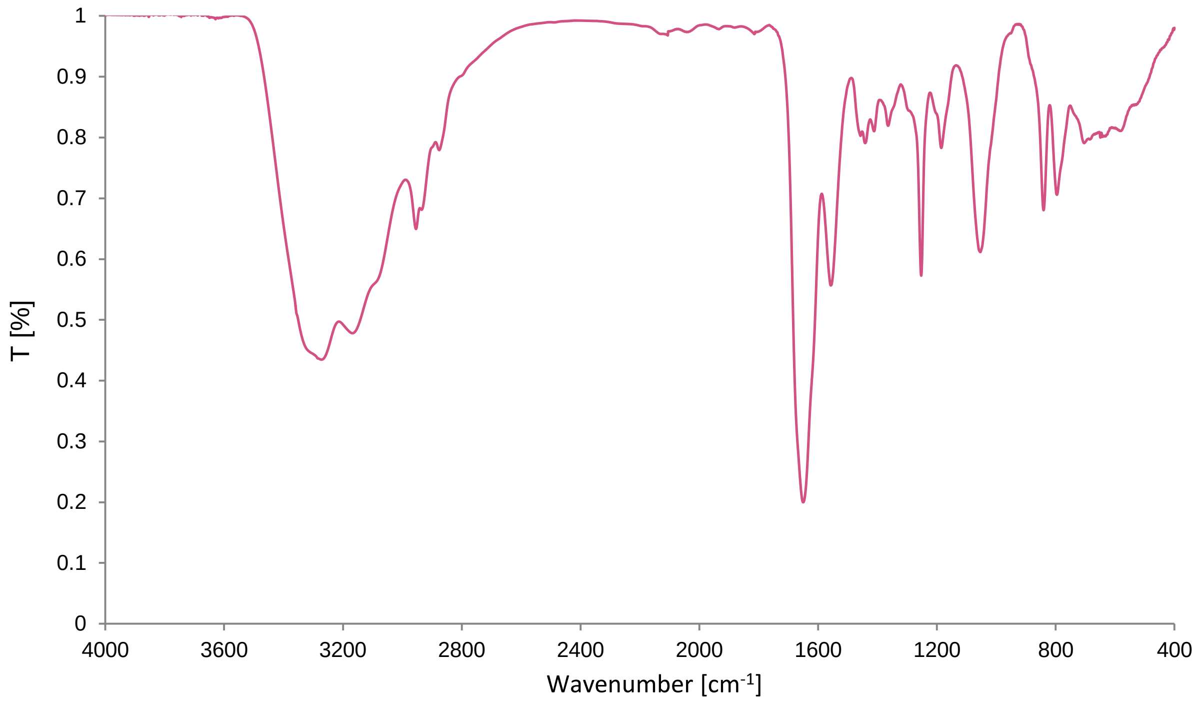

Figure S5.2.3. FT-IR spectrum of **2C** (**βALA**).

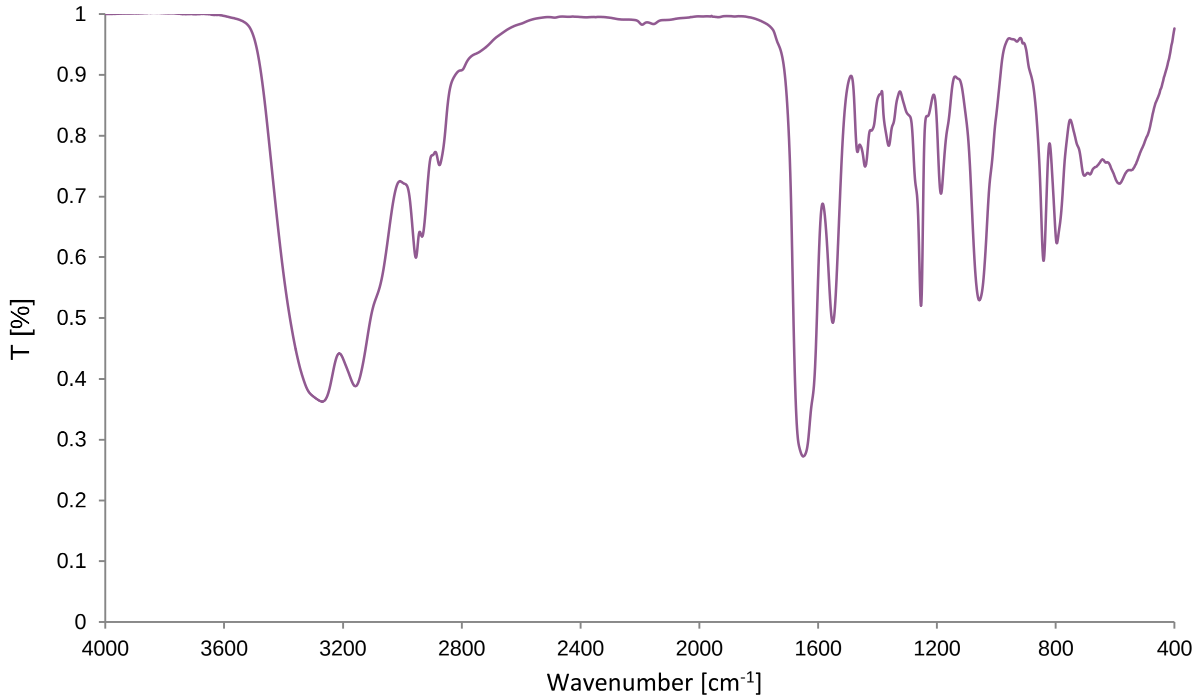

Figure S5.2.4. FT-IR spectrum of **3C** (**GABA**).

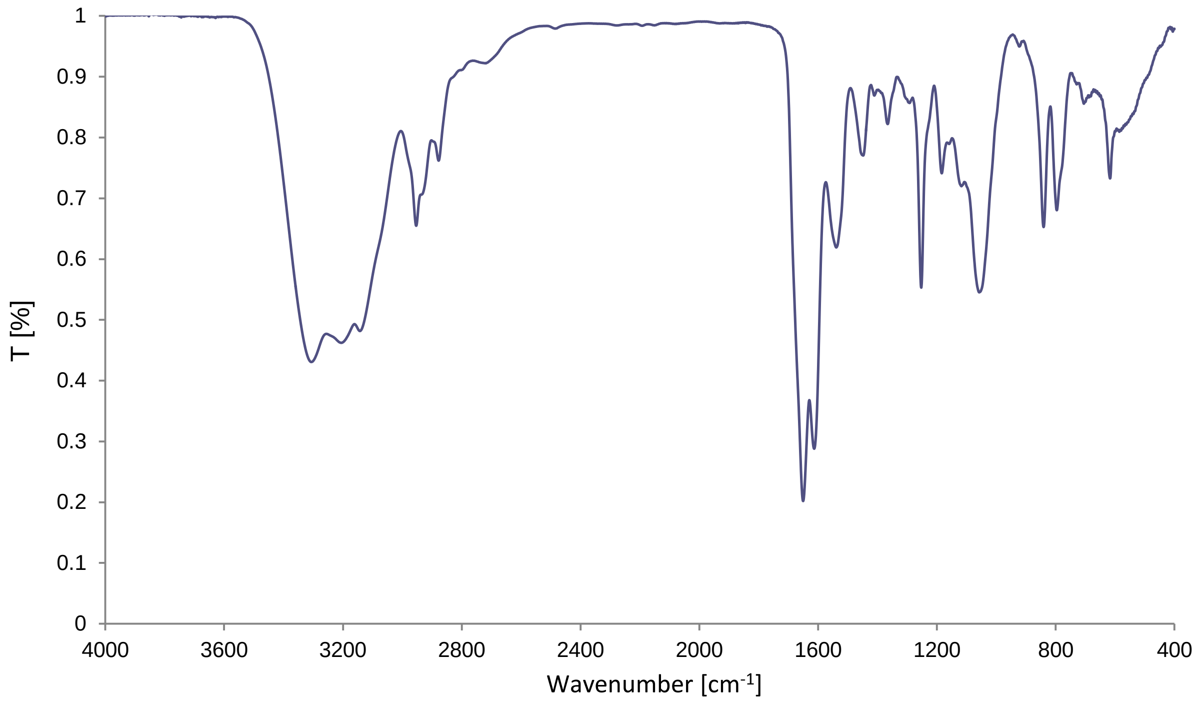

Figure S5.2.5. FT-IR spectrum of **4C** (**PRO**).

References

1. Tabisz, Ł., Tukibayeva, A., Pankiewicz R., Dobielska, M., Leska, B. Disiloxanes and Functionalized Silica Gels: One Route, Two Complementary Outcomes—Guanidinium and Pyridinium Ion-Exchangers. *PLoS ONE.* **10**(12), e0145680; 10.1371/journal.pone.0145680 (2015).
2. Babij, N. R. et al. NMR Chemical Shifts of Trace Impurities: Industrially Preferred Solvents Used in Process and Green Chemistry Organic Process Research and Development. *Org. Process Res. Dev.* **20**(3), 661-667 (2016).
3. Katrusiak A. Crystallographic autostereograms. *J. Mol. Graph. Model.* **19**, 363-367 (2001).
